# A genome-wide search of Toll/Interleukin-1 receptor (TIR) domain-containing adapter molecule (TICAM) and their evolutionary divergence from other TIR domain containing proteins

**DOI:** 10.1186/s13062-022-00335-9

**Published:** 2022-09-02

**Authors:** Shailya Verma, Ramanathan Sowdhamini

**Affiliations:** 1 National Centre for Biological Sciences, GKVK Campus, Bellary Road, Bangalore, 560065 India; 2 Institute of Bioinformatics and Applied Biotechnology, Bangalore, 560100 India; 3 Molecular Biophysics Unit, Indian Institute of Science, CV Raman Road, Karnataka 560012 Bangalore, India

**Keywords:** GWS, TICAM, TRIF, TRAM, TAG, TLR4, TLR3, Protein evolution

## Abstract

Toll/Interleukin-1 receptor (TIR) domains are cytoplasmic domain that mediates receptor signalling. These domains are present in proteins like Toll-like receptors (TLR), its signaling adaptors and Interleukins, that form a major part of the immune system. These TIR domain containing signaling adaptors binds to the TLRs and interacts with their TIR domains for downstream signaling. We have examined the evolutionary divergence across the tree of life of two of these TIR domain containing adaptor molecules (TICAM) *i.e.,* TIR domain-containing adapter-inducing interferon-β (TRIF/TICAM1) and TIR domain containing adaptor molecule2 (TRAM/TICAM2), by using computational approaches. We studied their orthologs, domain architecture, conserved motifs, and amino acid variations. Our study also adds a timeframe to infer the duplication of TICAM protein from *Leptocardii* and later divergence into TICAM1/TRIF and TICAM2/TRAM. More evidence of TRIF proteins was seen, but the absence of conserved co-existing domains such as TRIF-NTD, TIR, and RHIM domains in distant relatives hints on diversification and adaptation to different biological functions. TRAM was lost in *Actinopteri* and has conserved domain architecture of TIR across species except in *Aves*. An additional isoform of TRAM, TAG (TRAM adaptor with the GOLD domain), could be identified in species in the Mesozoic era. Finally, the Hypothesis based Likelihood ratio test was applied to look for selection pressure amongst orthologues of TRIF and TRAM to search for positively selected sites. These residues were mostly seen in the non-structural region of the proteins. Overall, this study unravels evolutionary information on the adaptors TRAM and TRIF and how well they had duplicated to perform diverse functions by changes in their domain architecture across lineages.

**Supplementary Information:**

The online version contains supplementary material available at 10.1186/s13062-022-00335-9.

## Background

Toll-like receptors play a major role in the innate immune system by recognizing diverse exogenous and endogenous biomolecules (viral RNA, Bacterial or self DNA, LPS, etc.) as their ligands and produces cytokines along with other inflammatory mediators during infections. Structurally they have an extracellular Leucine-rich repeat (LRR) domain, a transmembrane domain, and an intracellular Toll/Interleukin-1 receptor (TIR) domain [1]. The Toll-like receptor proteins identify the ligands by their extracellular LRR domain and then homo or heterodimerize to recruit the adaptor molecules like MyD88, TIRAP, TRIF (specific to TLR4 and TLR3) and TRAM (exclusive for TLR4 signaling pathway). The MyD88 & TIRAP dependent pathway ultimately releases pro-inflammatory cytokines, NF-ĸB, Tumor Necrosis Factor alpha (TNF-α), interleukin (IL-)1β, IL-6, and chemokines. TRIF & TRAM pathway releases both pro-inflammatory cytokines as well as anti-inflammatory mediator like Interferon Regulatory Factor 3 (IRF3), beta interferon (IFN-β), delayed NF-κB activation, type 1 IFN-α/β, IFN-α-inducible protein 10 (IP-10), MCP-5, RANTES, and nitric oxide [2].

**Fig. 1:**
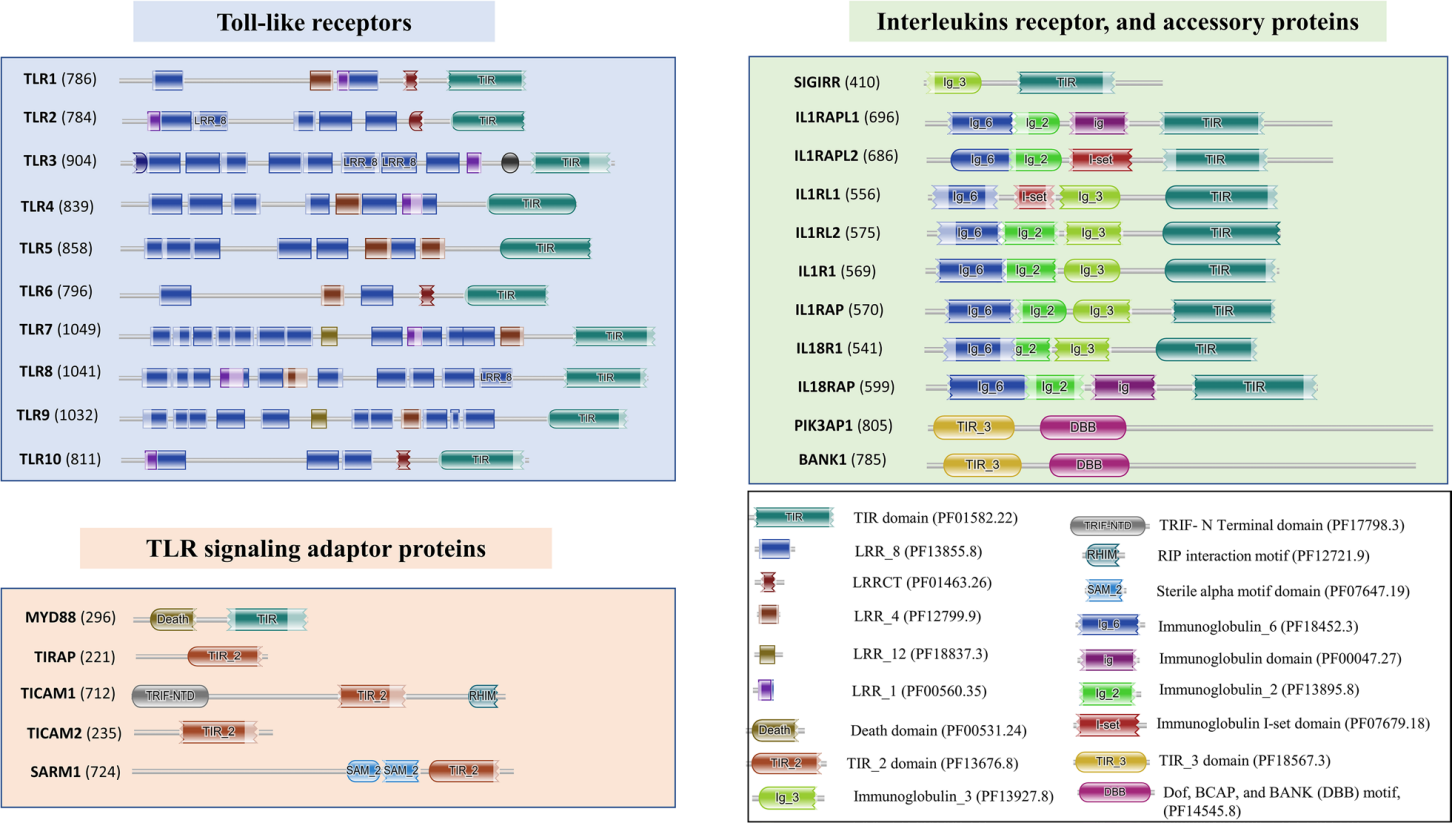
The domain architecture for TIR domain containing proteins from human are shown. Pfam ID for each of the domains are mentioned in the legend. The length of the protein is mentioned in the bracket. (TIR: Toll/Interleukin-1 receptor, LRR: Leucine rich repeat, LRRCT: C-terminal LRR domain)

Amongst these TLR proteins and its signaling adaptors, TIR domains are found in both. Apart from that, these domains are also present in Interleukins receptors as well as some accessory proteins. Overall there are 25 genes in the human genome that contain TIR domains as per PROSITE database—(Prosite ID: PS50104, Toll-like receptor proteins: TLR1, TLR2, TLR3, TLR4, TLR5, TLR6, TLR7, TLR8, TLR9, TLR10, TLR signaling adaptor proteins: MyD88, TIRAP, TRIF/TICAM1, TRAM/TICAM2, SARM1, Interleukins receptor, and accessory proteins: SIGIRR, IRPL1, IL1R1, ILRL1, ILRL2, IL18R, I18RA, IRPL2, PIK3AP1 and BCAP/BANK1). A figure depicting the domain architecture of these TIR domain containing proteins are shown in Fig. 1.

Amongst these TIR domain containing proteins, Toll-like receptors, MyD88, SIGIRR, and Interleukin receptor proteins share similar kind of TIR domain structure (Pfam ID: PF01582.22), but the adaptor proteins TIRAP, TRAM, TRIF and SARM1 share a different Pfam TIR_2 domain (PF13676.8). Besides these PIK3AP1 and BCAP have TIR_3 domain (PF18567.3).

These TIR domains are cytoplasmic in nature and consist of approximately 200 amino acids. They in general promote the assembly of signaling complexes via protein–protein homotypic or heterotypic interactions. The TIR structure contains a central five-stranded parallel β sheet surrounded by five helices. Although TIR domains from different proteins have similar structure, their amino acid sequence identity is less than 30% when compared amongst each other. This makes them significantly diverse in sequence and structure amongst Toll-like receptors (TLRs), TLR adaptors, and Interleukin receptors [3]. Sequence analysis has shown three highly conserved regions among the different family of TIR proteins: Box1 (FDAFISY), Box2 (GYKLC-RD-PG), and Box3 (a conserved W surrounded by basic residues) [4].

Multiple evolutionary studies are performed on the evolution of TLR family protein across vertebrates [5–7]. These phylogeny-based studies add to our understanding of the origin of TLR family proteins, their adaptors and probable signalling pathway. Evidence of **MyD88 in older taxa** explains the origins of MyD88 dependent pathways in invertebrates, although the TRIF and TRAM have resulted from an early duplication event in vertebrate TLR phylogeny [6]. This makes it interesting to mark the emergence of MyD88 independent pathways from the vertebrates. Another study showed the comparative and phylogeny analysis of TLR adaptors (TRIF, TRAM, and **TIRAP**) across 25 representative metazoans. The study aids to add knowledge about these adaptors and the evolution of their functional sites. Also, they have found shark to be the only non-Mammalia group to have TRAM [8].

All the previous evolutionary and phylogeny-based analyses were found to be focused around the TLR family and its divergence from the primitive organisms. Although the presence of the MyD88 adaptor molecules started in early invertebrates, it was interesting to study the origin of the MyD88 independent pathway. Early research suggests the emergence of the TIR domain-containing adapter molecule (TICAM) pathway among the basal chordates (Amphioxus) along with its structural and functional role [9]. Our study aims to look into the TLR MyD88 independent pathway adaptors (TRIF and TRAM) across all lineages. In this paper, we report genome-wide search for orthologs and analysis of domain architecture, sequence conservation, and evolutionary selection amongst those.

## Results

### Human TIR containing proteins and conserved motif

As described previously, TIR subfamily is represented at least in 25 genes in the human genome. They can be majorly classified into three categories: Toll-like receptor proteins (TLR1, TLR2, TLR3, TLR4, TLR5, TLR6, TLR7, TLR8, TLR9, TLR10), TLR signaling adaptor proteins (MyD88, TIRAP, TRIF/TICAM1, TRAM/TICAM2, SARM1), Interleukin receptors and accessory proteins (SIGIRR, IL1AP, IRPL1, IL1R1, ILRL1, ILRL2, IL18R, I18RA, IRPL2), PIK3AP1 and BCAP.

This set of proteins were further employed as queries to search for representative sequences from other taxa (Primates, Odd-toe ungulate, Even-toe ungulate, Carnivore, Placental, Whale and dolphins, Chiropteran, Rodentia, Lagomorpha, and Insectivores) to understand the phylogenetic relationships amongst these proteins. The sequences of the 25 TIR containing genes, along with their representative sequences from different taxa, are provided in Additional File 2.

A maximum likelihood-based tree is as Additional File 3. The respective branch lengths are mentioned and bootstrap values are shown in different sizes of circles. Also, the node ID shows the representative organism from each group. Each TIR domain containing proteins is colored distinctly. The phylogeny clearly shows the varying branch length and clustering of sequences into three major groups as specified earlier. The plasma membrane-based TLR proteins (TLR1, TLR2, TLR4, TLR6, TLR10) and endosomal membrane TLR proteins cluster separately (TLR3, TLR6, TLR7, TLR8, TLR9). TLR5 is found to cluster with TLR3 which may be because of closer identity amongst them. TRIF clusters with other adaptor molecules TRAM and TIRAP as the third cluster. Interestingly, in this phylogeny, the TRIF/TICAM1 was the protein with the highest branch lengths and appears within this cluster. From the motif search, TRAM and TRIF were seen to only have Common-4 motif conserved, unlike conventional TIR domain containing proteins which have Box1, Box2, and Box3 as well. Hence, we decided to perform genome-wide sequence search of TRAM and TRIF across all available taxa.

### Search for TRAM and TRIF orthologues

TRAM and TRIF play role in the MyD88 independent pathway and, are involved in IRF3 and IFN β signaling via TLR4 (involves both TRAM and TRIF) and TLR3 (only TRIF) receptors. Activation of TRIF dependent pathway also helps in dendritic cell maturation, thereby acts as a link between innate and adaptive immune responses [2]. Therefore, a query set of TRIF and TRAM proteins from 25 organisms across different orders of Mammalia were considered to search for their orthologs (please see “Methods” section).

**Fig. 2:**
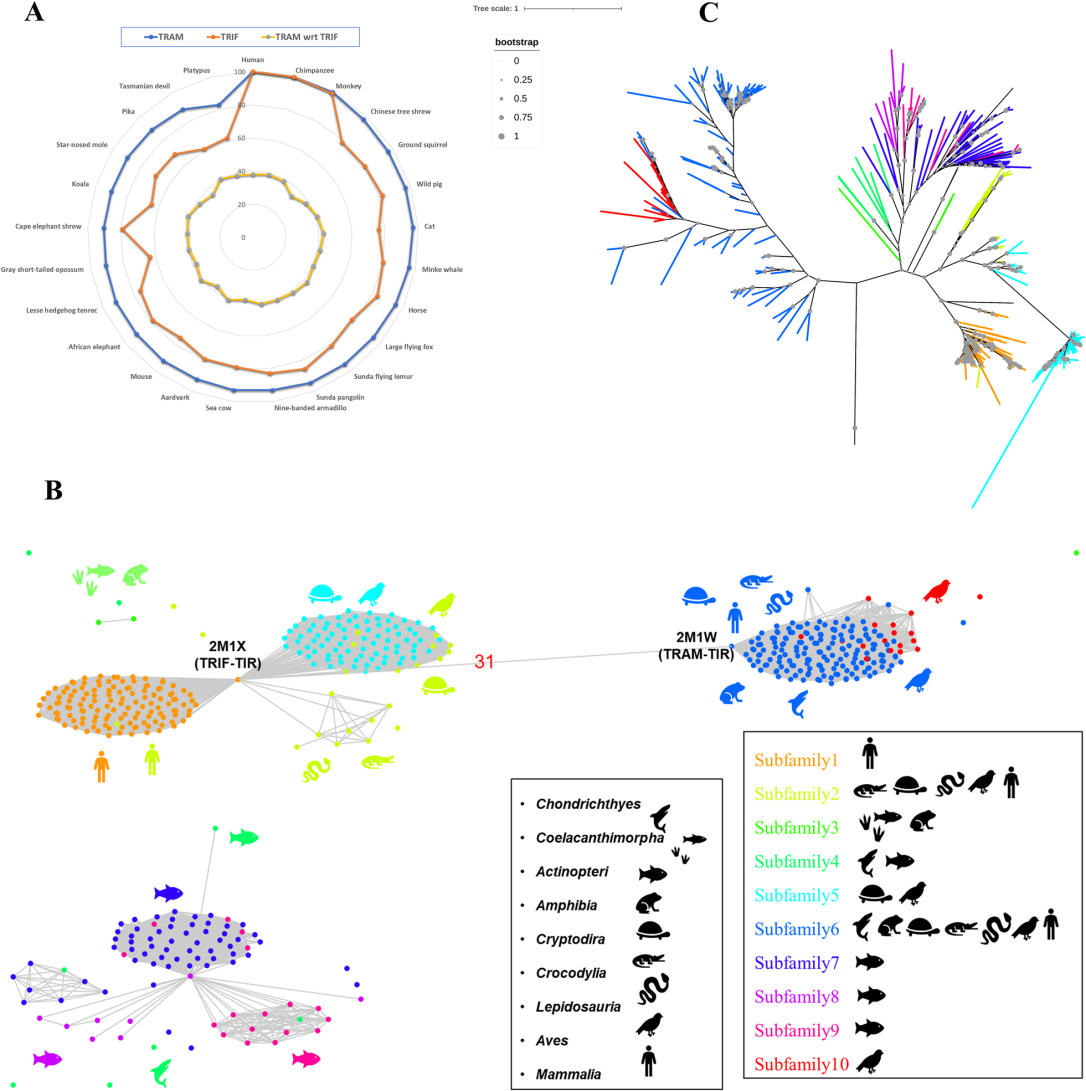
**A** The radar plot shows the sequence identity between the TIR domain of TRAM (blue) and TRIF (orange) of respective organism wrt human TRAM-TIR and TRIF-TIR. Also, the inner most line (yellow) represent sequence identity percentage of TRAM- TIR and TRIF-TIR within the same organism. **B** Sequence similarity network, with edges between 45 and 100% pairwise sequence identity, and clustering of sequences into subfamilies. Representative organisms from each taxon are represented across each subfamily. In the clustering method, TRAM protein separates distinctively from the other clusters. For the TRIF representatives, Actinopteri and Chondrichthyes separate as a distinct cluster. Mammals represents an individual cluster closer to clusters of Aves, Bifurcata, Crocodylia, Cryptodira, and Amphibia. Interestingly, members from the Amphibia clusters separates notably from the Reptiles clusters (Bifurcata, Crocodylia, Cryptodira). Thereby, explaining the extent of differences amongst these sequence in accordance with evolutionary perspective. **C** An unrooted phylogeny showing different subfamilies. The left cluster represents TRAM and right represents TRIF orthologs

TRIF and TRAM share higher sequence identity compared to other TIR containing proteins. So, for comparative analysis, a sequence identity comparison was done for the TIR domain of TRAM and TRIF which is shown in Fig. 2A. This shows TRAM-TIR shares around 40% sequence identity with TRIF-TIR across all Mammalia taxa. The orthologs obtained after CS-BLAST search from TRAM and TRIF queries were used to construct a subfamily-specific sequence similarity network (SSN) using ZEBRA which is shown in Fig. 2B [10]. The SSN show 10 distinct protein subfamily clusters represented by different colors. The nodes between 45 to 100% pairwise sequence identity was connected. Also, we found a pairwise sequence identity of only 31% across the TRAM-TIR (PDB ID: 2M1W) and TRIF-TIR (PDB ID: 2M1X) [11]. Additionally, Subfamily 6 and 10 were seen to be clustered together with TRAM-TIR thereby representing TRAM orthologs. Similarly, Subfamilies 1, 2, and 5 were clustering well with TRIF-TIR representing TRIF orthologs. Apart from these, Subfamily 3 and 4 were found to be scattered and Subfamily 7, 8, and 9 clustered together. Few sequences were considered as outliers and were not shown in SSN. We constructed an unrooted phylogeny from CSBLAST hits to examine the relationship between these bigger clusters. The unrooted phylogeny is shown in Fig. 2C and it shows well-separated clades diverging at the base of the tree. The basal node represents sequences from Belcher's lancelet (*Branchiostoma belcheri*) and Florida lancelet (*Branchiostoma floridae*)*,* commonly referred as Amphioxus and is grouped together as *Leptocardii*. These are known to be the oldest basal chordates with MyD88-independent pathway [9]. A similar pattern of clustering is seen in the phylogeny tree and TRAM separates well and distinctly from TRIF. Further, since subfamilies 7, 8, and 9 cluster with TRIF, a detailed analysis of genes that harbour these domains were performed. Analysis of domain architecture can enable to understand gain and loss of co-existing domains and diversification of function.

### Domain architecture among subfamilies

A typical human TRIF protein has TRIF-NTD, TIR_2, and RHIM domains. Here the N-terminal is used for activation of IFNβ promoter activity [12]. TIR_2 domain is involved in homo and heterotypic TIR interaction for signaling and the RHIM domain is important for NF-κB activation [13]. Whereas the TRAM protein contains only the TIR_2 domain. Additionally, TRAM’s isoform, TAG contains EMP24_GP25L along with the TIR_2 domain. This EMP24_GP25L domain is implicated in bringing the cargo forward and binding to coat protein [14].

**Fig. 3:**
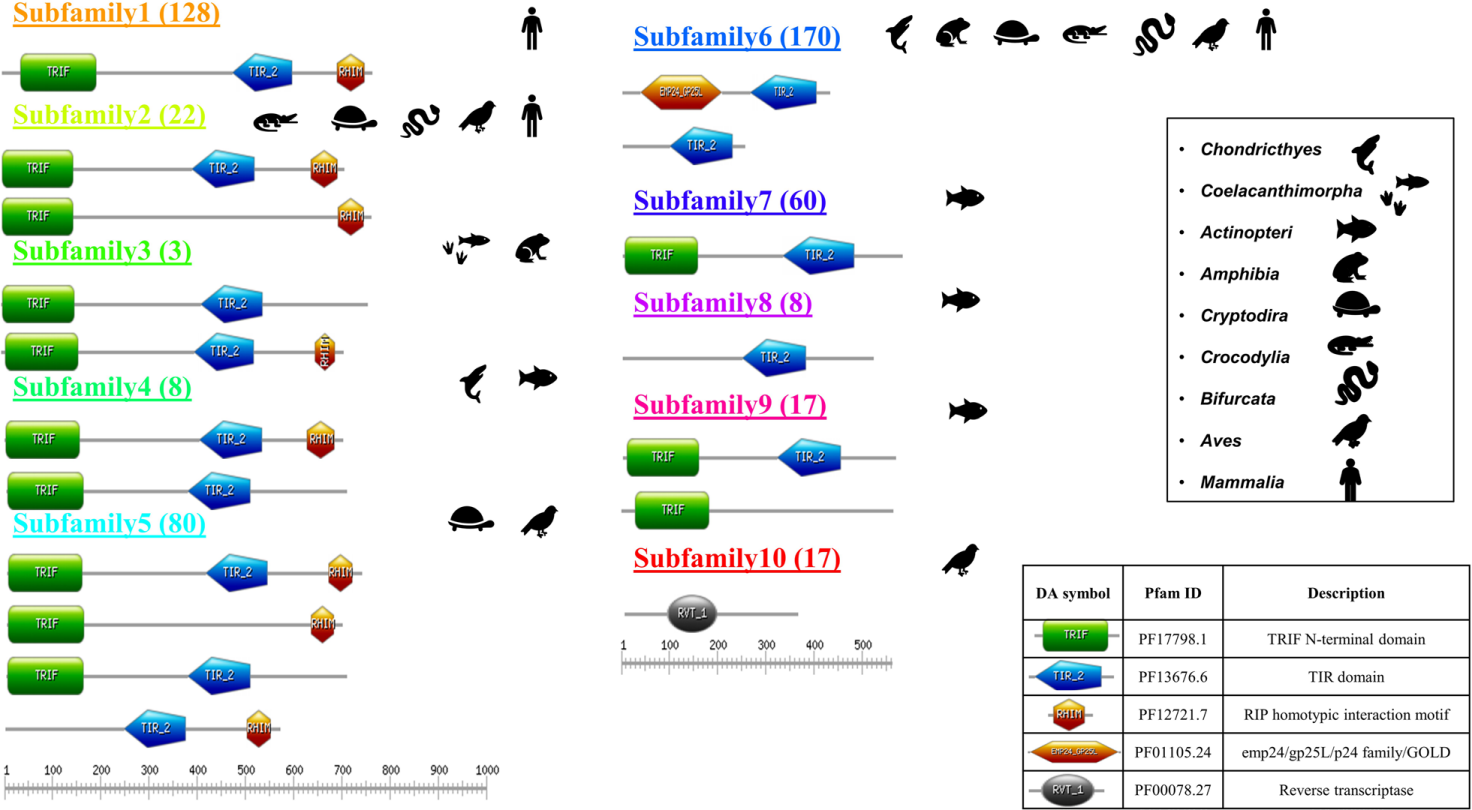
The picture shows different subfamily and corresponding unique domain architecture present in them. Also, the representative taxa of the cluster are shown as cartoon icons. The PfamID and description for each domain is also mentioned

Next, we examined the unique domain architectures and the representative taxa amongst them. A pictorial representation of the same has been shown in Fig. 3. Also, the number of sequences in each category is mentioned. Thereby with respect to Subfamily clusters as obtained in SSN analysis, Subfamily 6 and 10 includes TRAM and TAG which is a splice variant of TRAM (TRAM adaptor with the GOLD domain) [15]. Also, subfamily 10 majorly consists of hits from Aves, and in which no conventional TIR domain was found instead a RVT_1 (Reverse transcriptase domain family) domain was found. Apart from this Subfamily 1, 2, and 5 consists of TRIF sequences with well-annotated domain architecture from *Mammalia, Aves, Bifurcata, Crocodylia,* and *Cryptodira*. Amongst these Subfamily 1, 2, and 5 each of them clusters distinctively, with Subfamily 1 including sequence only from *Mammalia.* Whereas Subfamily 5 and 2 both have sequences from *Aves and Cryptodira*. Moreover Subfamily 2 also have sequences exclusively from *Bifurcata and Crocodylia.* Besides these, Subfamilies 3 and 4 have sequences from *Chondrichthyes, Coelacanthimorpha* (living fossil), and few sequences from *Amphibia* and *Actinopteri*. But most members of *Actinopteri* clusters together under Subfamily 7, 8, and 9. The domain architecture for *Actinopteri consists* of TRIF, TIR_2 domain and lacks the RHIM domain (HMM scan, inclusion e-value = 0.001).

Sequences from *Leptocardii* were considered as an outlier, so we looked at the secondary structure of sequences from the primitive organisms. Sequences from *Leptocardii, Chondrichthyes,* and *Coelacanthimorpha* were compared with respect to sequences from human TRAM and TRIF. Good conservation along the TIR domains can be seen in the alignment. The image is attached in Additional File 1: Fig. S3. Moreover, good secondary structure conservation hints towards ancestral homology.

### Phylogeny of TRAM and TRIF orthologs

We separated the hits into TRAM, TAG, and TRIF subgroups and constructed a phylogeny using Maximum likelihood with 100 bootstraps and branch lengths.

TRIF orthologs were found across *Chondrichthyes, Coelacanthimorpha, Actinopteri, Amphibia, Cryptodira, Crocodylia, Bifurcata, Aves,* and *Mammalia*. Human TRIF has three typical and two atypical TRAF6 binding sites. The generic motif representing this site is denoted by [PxExxD/W/E/F/Y]. The motif sequence and residue positions of human TRIF protein for typical TRAF6 binding sites are PEEPPD (86–91), PEEMSW (250–255) and PVECTE (301–306), whereas the same for atypical sites are PLESSP (491–496) and PPELPS (264–269). Amongst these, the second typical TRAF6 binding site [PEEMSW (250–255)] is one of the most important sites for TRIF interaction with TRAF6 and NF-κb induction [16]. Apart from these, TRIF also has a pLxIS motif ([[NDQEKR]LxIS]) which is a phosphorylation site used for inducing IRF3 activation and RHIM interacting motif ( [[IV]Q[ILV]GxxNx[MLI]]) which is part of RHIM domain and is important for inducing apoptosis [17, 18]. Human TRIF has these motifs in following order with their residue position ~ TRAF6(86–91)~**pLxIS(207–210)**~**TRAF6(250–255)**~atypical-TRAF6(264–269)~TRAF6(301–306)~atypical-TRAF6(491–496)~**RHIM motif(687–695)**. Amongst these, the highlighted motifs are shown in the detailed phylogeny in alignment form in the same order as they appear in human TRIF protein (Uniprot ID: Q8IUC6) [12]. Members of *Mammalia, Amphibia, Cryptodira, Coelacanthimorpha* shows conserved pLxIS motif and among *Bifurcata*, *Actinopteri, Aves, Crocodylia* only few organisms have pLxIS motifs. Besides these, the second typical TRAF6 binding sites (PEEMSW) is seen conserved only in *Mammalia* and some organisms of *Aves*, it may be possible that other TRAF6 binding sites may be functional in other taxa. Whereas the RHIM motif along with the RHIM domain are seen to be conserved in all taxa except for *Actinopteri* and *Leptocardii*.

We have represented motifs and domain annotation for each organism in the phylogeny. We observe that none of the members of *Crocodylia* taxa show TIR_2 domains with inclusion e-value (HMM scan, inclusion e-value = 0.001). Even on increasing the value to 0.1 only one of the members *Crocodylus porosus*, shows TIR domain at e-value = 0.048, but that was an insignificant search (Fig S6). The detailed phylogeny with motifs and domain annotations is attached as Additional File 4.

Unlike TRIF, human TRAM (Uniprot ID: Q86XR7) consists of a putative *N-Myristoylation site* (residue position: 2–7) that helps in its localization to the plasma membrane and is critical for TLR4 pathways in response to LPS [12, 19]. The *N-Myristoylation site* has a consensus motif sequence of [G{EDRKHPFYW}xx[STAGCN]{P}], which is important for signal transduction [20]. TRAM protein also has a putative TRAF6 binding motif [PxExxP] (residue position: 181–186) that helps to interact with TRAF6 and mediate activation of the inflammatory responses by TLR4 [21]. Upon observing these in the phylogeny, we found the TIR_2 domain annotation and conserved motifs were missing from *Aves*, suggesting they may not have a functional TRAM protein. Additionally, *Aves* taxa has the highest branch lengths and highest amino acid distances as shown in Additional File 5. This implicates that *Aves* taxa may have undergone a higher divergence and genetic changes over evolutionary time. Previously reported studies with representative sequences across taxa claims TRAM to be lost in fishes, birds, and amphibians. But interestingly by our genome-wide search, we found TRAM orthologues for Aves [22]. None of the TRAM orthologue sequences were retrieved from *Actinopteri* (bony fishes).We found TRAM ortholog sequence in *Callorhinchus mili* from Chondrichthyes (cartilaginous fish), it also shows a concentional TIR_2 domain architecture along with conserved motif. This may be the oldest organism with functional motifs conserved with TIR_2 domain. Also, amongst members of *Amphibia*, all the hits have conserved TIR domain along with conventional *N-Myristoylation* motif except for *Xenopus laevis*. On combining all of our observation we observe that TRAM may have diverged from TRIF and first appeared in *Chondrichthyes* (399 mya), was then lost in *Actinopteri,* and then reappeared in *Amphibia* (323 mya), *Cryptodira, Crocodylia, Bifurcata, Aves*, and *Mammalia.*

**Fig. 4:**
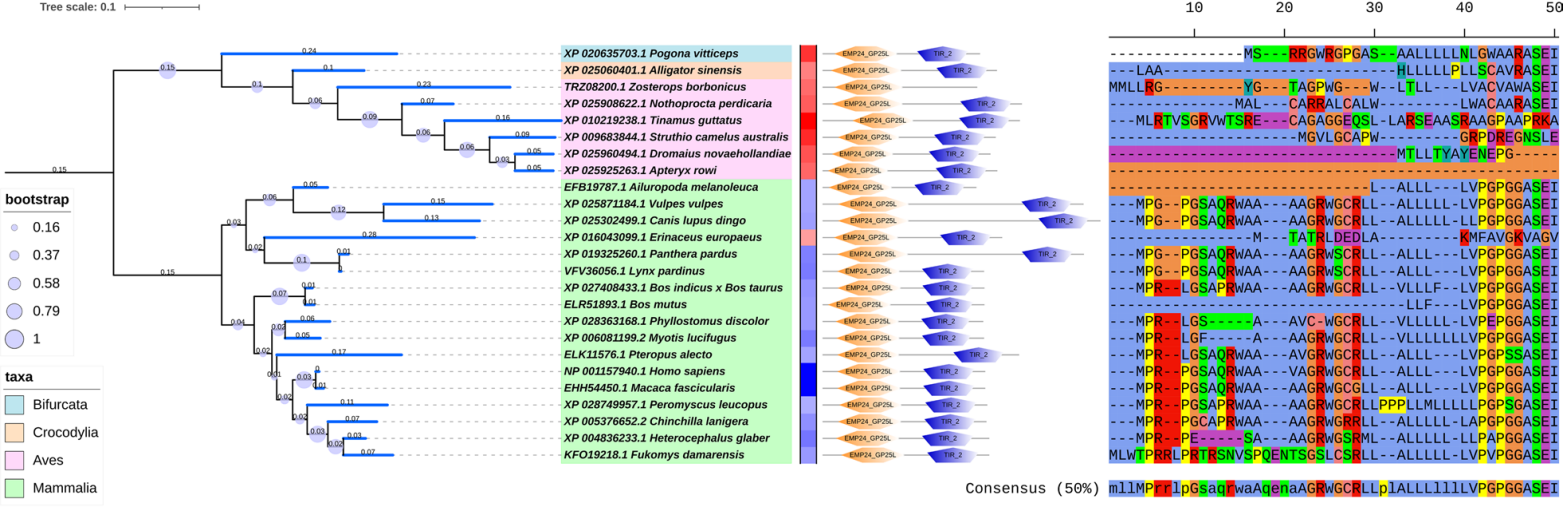
A detailed phylogeny of TAG from different organisms, along with amino acid distance wrt *Homo sapiens*, domain architecture, and alignments across initial positions 1–20. The branches were colored to display the subfamily it belonged to and node IDs were colored based on the taxa group. A relative amino acid distance was shown with respect to Homo sapiens sequences by color gradient boxes (blue representing the minimum distance, white being the midpoint gradient, and red being the maximum distance). Also, the domain architecture was shown for each sequence. Besides these conserved motifs were shown in the order of their occurrence in an alignment form next to each node of the phylogeny

TRAM isoform (Uniprot ID: Q86XR7-2) with GOLD domains was also seen across one *Bifurcata*, one *Crocodylia*, a few *Aves,* and *Mammalia* taxa. All of them depicted the well-conserved domain architecture of EMP24_GP25L (primarily involved in the transport of cargo molecules from the endoplasmic reticulum to the Golgi complex [23]) and TIR_2 domain. The difference in sequences of canonical human TRAM and isoform TAG is seen at the position from 1 to 20 (MGIGKSKINSCPLSLSWGKR→MPRPGSAQRW..), this has been shown in the alignment. A similar pattern can be seen across *Mammalia.* TAG seems to be found mainly in *Mammalia, Aves*, and in a few cases of *Crocodylia*, and *Bifurcata*. Extensive genome sequencing can help recognize TAG across other organisms. An extensive phylogeny showing the presence of TAG in different taxa is shown in Fig. 4.

**Fig. 5:**
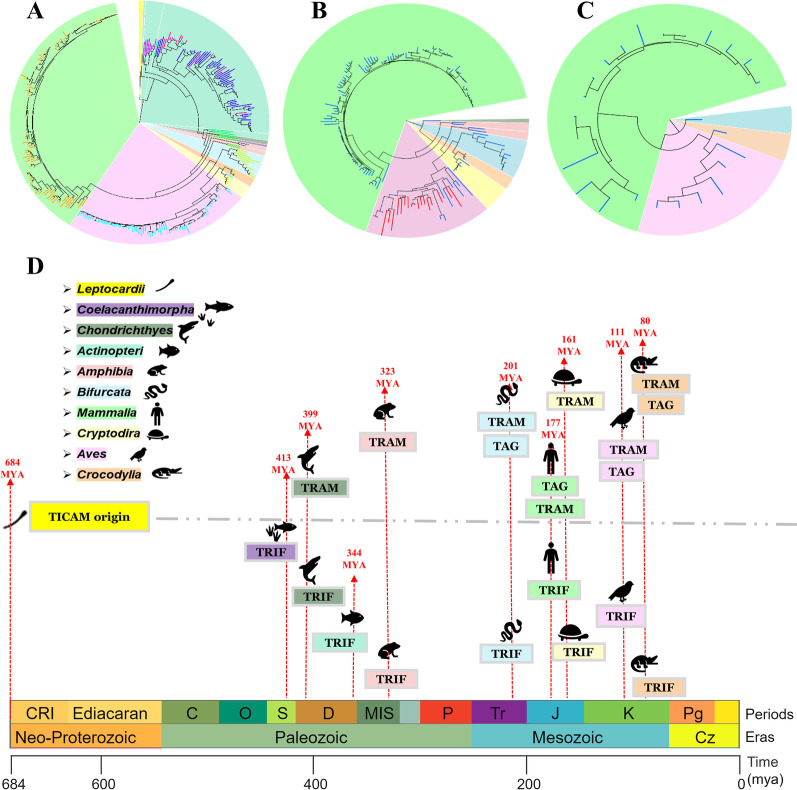
The figure in the top panel shows a representative phylogeny for TRIF (**A**), TRAM (**B**) and TAG (**C**) respectively. The colored region represents organisms from different taxa and the node colour represents previously categorised ZEBRA subfamily. **D** This panel represents the evolutionary timeline from TICAM origin from Leptocardii and divergence into TRIF, TRAM and TAG. Here, TRAM was seen to be lost in Coelacanthimorpha and Actniopteri. The time period for the organisms were taken from Time Tree considering divergence between the hits. The order of the Eras with their Periods are Neo-Proterozoic Era (Cryogenian and Ediacaran), Paleozoic Era (Cambrian, Ordovician, Silurian, Devonian, Mississippian, Pennsylvanian, Carboniferous, and Permian), Mesozoic Era (Triassic, Jurassic and Cretaceous) and Cenozoic Era (Paleogene, Neogene and Quaternary)

A representative phylogeny from each category is shown below showing the proportion of organisms. The evolutionary timeline for the divergence of TICAM is also shown below in Fig. 5 [24]. The detailed phylogeny for TRIF and TRAM is attached as Additional Files 4 and 5.

### Conservation of Synteny

To focus on the orthologous relationships of TRIF and TRAM, we next examined the syntenies and check the preservation of the order of genes amongst the species and check for the neighbour genes. One representative from each taxon e.g., *Homo sapiens (Mammalia), Falco peregrinus (Aves), Gekko japonicus (Bifurcata), Alligator mississippiensis (Crocodylia), Chelonia mydas (Cryptodira), Xenopus laevis (Amphibia), Callorhinchus milii (Chondrichthyes), Danio rerio (Actinopteri), Latimeria chalumnae (Coelacanthimorpha)* and *Branchiostoma belcheri (Leptocardii)* were selected*.* The accession ID and domain architecture for these representatives can be seen in Additional Files 4 and 5.

**Fig. 6:**
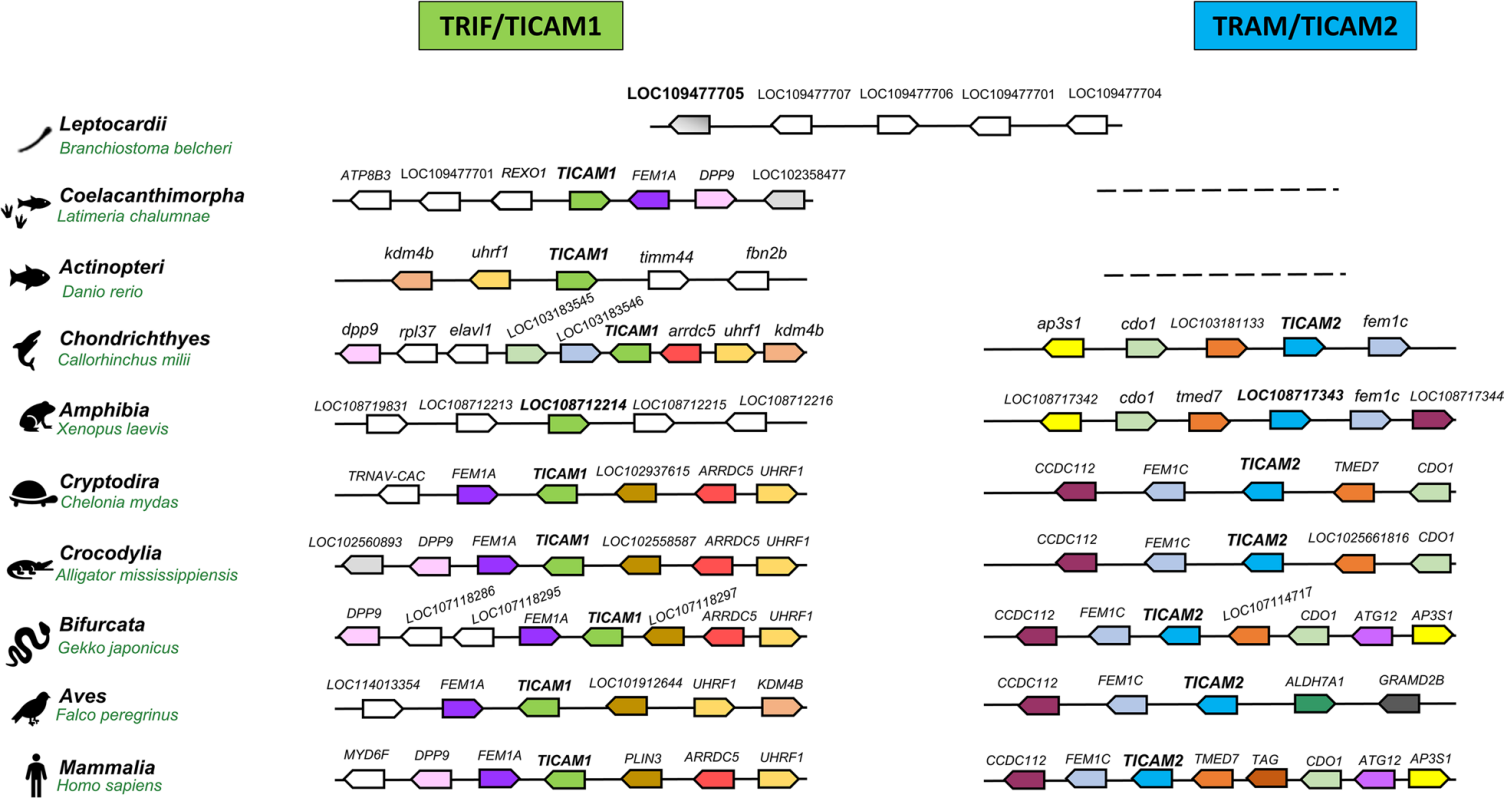
Gene synteny for TRIF and TRAM protein from different taxon

There is overall good correspondence amongst the neighbours of TRAM and TRIF orthologs, except TRIF of *Amphibia* (lacks the gene neighbours), and TRAM of *Aves* (does not have a full one-to-one correspondence) as seen in Fig. 6. Interestingly, FEM1C and CDO1 like genes were also found to be neighbouring TICAM2 genes in *Chondrichthyes* and other TICAM2 orthologs. Due to the event of whole-genome duplication occurring at *Actinopteri*, two forms of TICAMs are generated. Since these duplicates are preserved across the lineages, there may be subfunctionalization or neofunctionalization [25]. To ascertain this, it will be interesting to perform functional characterization of distant orthologs from *Coelacanthimorpha* and *Actinopteri*.

### Evolutionary selection pressure in TRIF and TRAM

From the previously obtained TRIF and TRAM phylogeny we observed the variation among branch lengths, and also the varied domain architecture amongst the sequences.

**Fig. 7:**
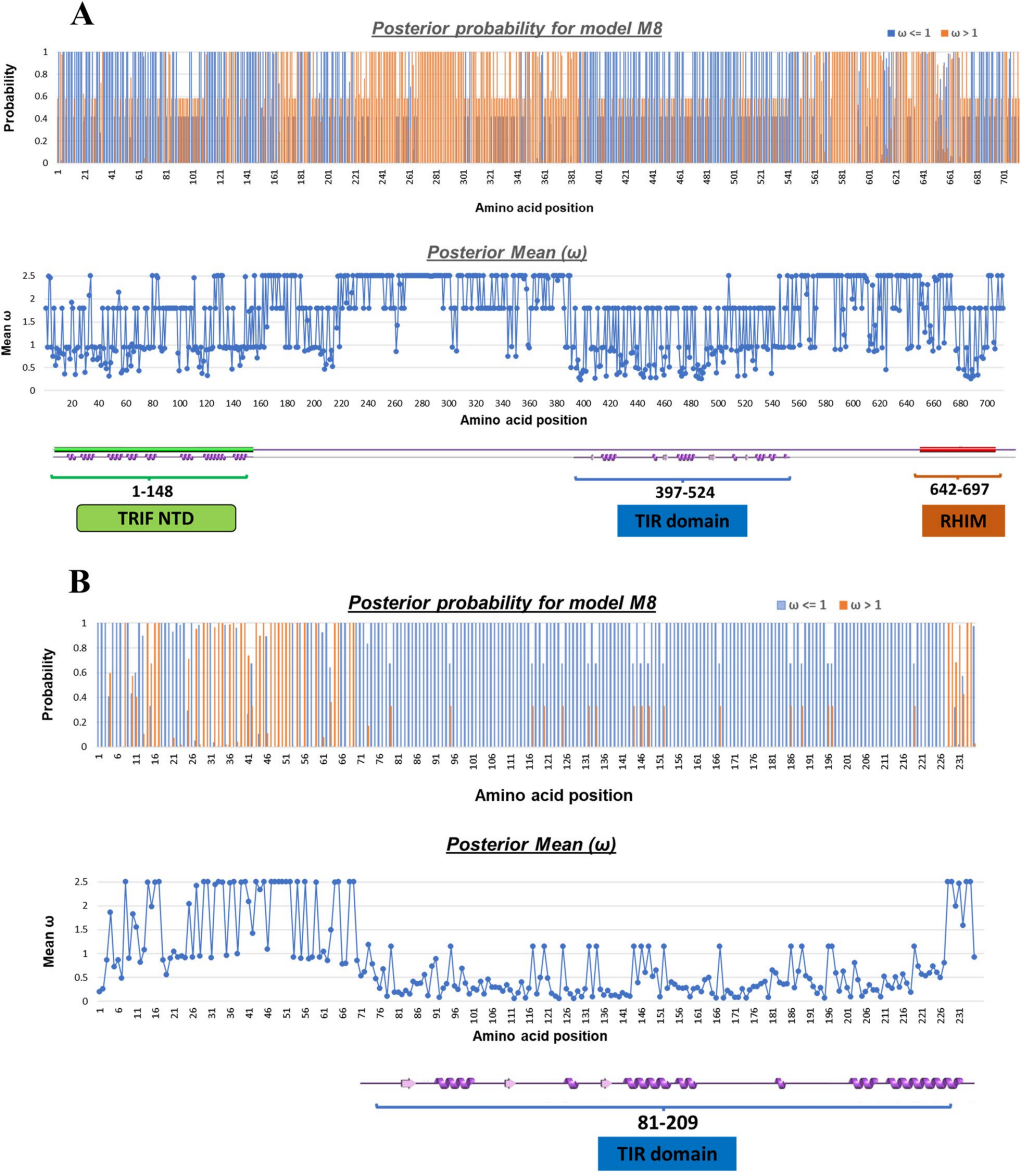
The plots show two graph (i) posterior probability obtained from model M8 along the amino acid of the protein. Here ω <  = 1 is shown in blue and ω > 1 is shown in orange. (ii) Shows the mean ω along the amino acid of the protein. **A** TRIF protein **B** TRAM protein

We were further interested to look into the selection pressure of TRIF and TRAM protein. We used the orthologues sequences and used site model from Codon substitution models (codeml) of PAML4.9 package. The implemented site models (M0, M1, M2, M3, M7, and M8) allowed ω ratio (ω = d_N_/d_S_, ratio of nonsynonymous/synonymous substitution rates) to vary among codon sites in protein. Based on the Likelihood ratio test, using the Bayes empirical Bayes (BEB) method or posterior probabilities of model **M8** for 11 site classes (k = 11) along the sequence of the protein was plotted. The values from 11 site classes were grouped into two categories as ω > 1 and ω < 1. Also, another graph depicting the mean probabilities for each site was plotted in Fig. 7.

From both these sequences, one can notice that the posterior probability of ω was moreover <  = 1 for sequences in domain regions. Although some positively selected sites were detected. The propensity of these positively selected sites was high and mostly amongst the non-structural regions. A list of positively selected sites with a probability above 0.95 has been provided in the Additional File 1: Figs. S4 and S5.

## Discussion

The role of Toll-like receptors is important for innate immunity and these adaptors, apart from the conventional (MyD88 and TIRAP) ones, provide an alternate pathway for the production of inflammatory mediators. Previous studies have concentrated on the evolution of TIRs and adaptors involved in the MyD88-dependent pathway. However, the evolutionary lineage of adaptors that are known to operate in MyD88-independent pathway have not been studied in detail. The prime objective of our study has been to look into the divergence of TICAM to TRIF and TRAM. Unlike the MyD88 pathway, which seems to have emerged in early invertebrates, TRIF and TRAM related pathway trace back and emerge with the duplication event in vertebrates. A previously reported study has shown these adaptors in 25 metazoans [8], but details about the evolution of these proteins is lacking.

We performed our sequence searches in whole genomes, by employing all the TIR domains in the human genome as our queries. We later extended the searches to include TRIF and TRAM adaptors and accumulated their orthologues in our study. As expected, the conservation of Box1, 2, and 3 were found in these proteins, apart from that an additional motif was seen to be conserved. This was named Common4 and was the only motif present in TRIF and TRAM. This makes these adaptors peculiar to be studied. From the sequence-based phylogeny of the orthologs, *Leptocardii* appears as the oldest ancestor for these proteins. This was referred as TICAM in basal chordates for the MyD88 independent pathway [9]. Although they do not seem to have conserved domain structures or motifs responsible for signaling like *Homo sapiens.* Further, with our sequence search approaches, we found TAG (an isoform of TRAM with GOLD domain) which is seen in some species of *Bifurcata, Crocodylia, Aves,* and *Mammalia.* We also observed, through synteny analysis, that TRAM lineages and their immediate gene neighbours to be more highly conserved, as compared to TRIF where some ambiguity was seen for *Actinopteri* and *Amphibia.* TIR domains within TRAM are more conserved than in TRIF (Fig. 2a) and the variety of domain architectures are more in TRIF (Fig. 3).

Additionally, to extend the residue-based study on the full-length sequence of the protein, we performed a site model-based analysis to detect positively selected residues. Amino acid pertaining to non-domain regions were found to be positively selected in both the protein family. Overall, this study helps us in understanding a little more about these adaptors and their evolution (Additional files 6, 7, 8, 9, 10, 11).

Based on the presence of these adaptors amongst different taxa, we can explain the signaling of TLR3 and TLR4 based immune pathways. The presence of TRIF aids to boost the endosomal TLR3 pathway by recognizing double-stranded RNA, a major form of genetic information carried by viruses. Whereas the TRAM’s presence along with TRIF can tell us about the functioning of the endosomal TLR4 pathway. The evidence of TRAM from *Chondrichthyes* makes us wonder about the need for the endosomal pathway of TLR4. This study can be further extended by a comparative analysis of orthologs among interacting partners of the signaling pathways. It will be interesting to track the presence or function of TRIF related inflammatory mediators, like IRF3 and IFN, in the older taxa. A structural determination for TICAM from ancestral taxa will help us know better about its function.

A potential limitation of the study would be the absence of high-quality data for ancestral taxa. Also, among groups like *Crocodylia, Amphibia,* and *Cryptodira,* whole genome sequence information is available only for a few species. To elucidate a taxa-specific evolutionary pattern and comment on group-specific evolution, we need to accumulate more data from multiple organisms. This will become better and more feasible with increasing numbers of whole genome sequencing of non-model organisms.

## Conclusion

The current study is aimed at a systematic search and survey of TICAM orthologs in all the available genomes. We examined the domain architecture of genes that bear these domains and map the TICAM divergence to TRIF and TRAM across timescales. We also found evidence of the isoform of TRAM, TAG, and its presence is dated around 201 mya. Analysis of conserved, co-evolving residues and codon-based analysis was performed to identify positively selected sites amongst orthologs.

TRAM domains play important role in TLR4-mediated endosomal MyD88 independent pathway, and TRIF is the sole adaptor domain for the TLR3 signaling pathway involved in ds RNA recognition. Therefore, this study will help us know more about the immune systems in older taxa and how they evolved during evolution.

## Methods

### Query dataset and sequence search

We initially curated the human TIR domain sequences from Uniprot and filtered the hits to include Swiss-Prot reviewed sequences. Further, amongst these, those which followed PROSITE-ProRule annotation: PRU00204, were only selected. This PROSITE profile is specific to the pattern of TIR domains’ scaffold that promotes assembly of signaling complexes via protein–protein interactions. Motif search was performed using MEME suite by classic search method and gapped local alignment was performed by the GLAM2 module using protein seqeuences as input structure [26–28]. Such human TIR sequences were used as a query to search for best homologs using BLAST [29] with an e-value of 10^–10^. Best representatives of Primates, Odd-toe ungulate, Even-toe ungulate, Carnivore, Placental, Whale and dolphins, Chiropteran, Rodentia, Lagomorpha, and Insectivores were selected for each query. The motif search was extended to this set of proteins (275 proteins).

In order to search the homologues of TRAM and TRIF, we enriched our dataset to include organisms from 22 orders across the Mammalian class. TRAM and TRIF proteins of different organisms from the following orders were included. Monotremata (*Ornithorhynchus anatinus*), Didelphimorphia (*Monodelphis domestica*), Dasyuromorphia (*Sarcophilus harrisii*), Diprotodontia (*Phascolarctos cinereus*), Cingulata (*Dasypus novemcinctus*), Proboscidea (*Loxodonta africana*), Afrosoricida (*Echinops telfairi*), Tubulidentata (*Orycteropus afer afer*), Rodentia (*Marmota flaviventris*), Primates (*Pan troglodytes*), Eulipotyphla (*Condylura cristata*), Chiroptera (*Pteropus vampyrus*), Artiodactyla (*Sus scrofa*), Cetacea (*Balaenoptera acutorostrata scammoni*), Perissodactyla (*Equus caballus*), Carnivora (*Felis catus*), Lagomorpha (*Ochotona princeps*), Macroscelidea (*Elephantulus edwardii*), Scandentia (*Tupaia chinensis*), Dermoptera (*Galeopterus variegatus*), Sirenia (*Trichechus manatus latirostris*), Pholidota (*Manis javanica*) orders along with two model organisms (*Mus musculus, Macaca mulatta*) protein and *Homo sapiens*.

Later, the TRAM and TRIF protein from 25 organisms were used as a query to perform CS-BLAST [30] against the NR_Sept2019 database. The search was done using a python script to include all sequences individually with a very stringent e-value of 10^–10^ and up to 5 iterations after which it got saturated. The results from all CS-BLAST searches were combined and using an in-house script the output of CS-BLAST was converted into a tabular format. These results were further filtered using a query coverage filter of more than or equal to 50% and a sequence identity filter of more than or equal to 30% (keeping in mind the Twilight zone of protein sequence alignment) [31]. The list of hits obtained from multiple query search after considering the query coverage and sequence identity cut off for TRIF and TRAM orthologues are shown in Additional Files 14 and 15 respectively. The sequence of reference ID from these hits was retrieved using blastdbcmd module of BLAST version 2.9.0+. To remove the redundancy amongst sequences, CD-HIT was used to cluster the hits with 100% identity cutoff [32]. These hits were further divided into subfamilies based on clustering pattern by constructing sequence similarity network (SSN). These classifications were done using ZEBRA2, based on the CD-HIT clustering approach with a sequence identity threshold of 30% [10]. Based on subfamily clusters and phylogeny, proteins homologous were categorized into TRAM and TRIF family. Here we also used the protein sequence from PDB entries 2M1X and 2M1W, that corresponds to TIR domain region from TRIF and TRAM human protein respectively. We used these TIR sequence along with the full-length TRIF and TRAM orthologs sequence to see where does the TIR sequence clusters in the SSN.

### Domain annotation

Domain architectures were searched for these sequences using Hmmscan modules from the HMMER suite package (version 3.1b2) against Pfam database (version 31.0) [14] with an e-value of 0.01 and inclusion threshold of 0.001. The output for domain architecture was generated using a python script. Sequences, which connected to the Pfam entry, TIR_2, were alone considered as true positives. Those sequences which connected to Pfam domains, TRIF-NTD, TIR_2, and RHIM domains were pooled as TRIF homologues. The domain architecture of each ortholog with the domain boundary and e-value are shown in Additional File 13. The domain architecture diagram was made using My Domains from Expasy [33]. For Fig. 1, the domain architecture were taken from the HMMER webserver, by using hmmscan with e-value cutoff of 0.01 [34]. Sequences apart from these were looked at manually concerning their secondary structure and alignment profile. The secondary structure predictions were performed using PSIPRED [35]. Also, Ali2d scan was used for multiple secondary structure prediction and its results were visualized in 2dSS alignment viewer [36, 37].

### Phylogeny tree construction

The fasta sequences were aligned using MUSCLE v3.8.31 [38] and a phylogenetic tree was constructed using the maximum likelihood method with 100 bootstraps in MEGAX [39]. The evolutionary timelineof the taxa were calibrated using TimeTree [24] based timeline for divergence of nodes. Amino acid distances were also calculated in MEGA using the p- distance method with uniform rate among sites, the homogenous rate among lineage, and pairwise deletion of gaps. Relative rate test was performed for sequences with higher branch lengths using Tajima relative rate test in MEGAX. This was calculated using ‘Homo Sapiens’ as a reference and the oldest descendant species as an outgroup to confirm if organisms with higher branch length evolve at the same rate or reject the null hypothesis. These data were added to the phylogeny and visualized using iTOL [40].

### Conservation of Synteny

Genome assembly for one representative organism from each taxon was used to look into the gene neighbours. The sequence ID corresponding to TRAM and TRIF protein were searched in NCBI and the neighbours were found using the Genome Data Viewer [41].

### Strength of evolutionary selection

Nucleotide codon sequences were retrieved for each protein ID using the Batch Entrez mode of NCBI [42]. They were further aligned using MUSCLE and then converted to codon alignment using PAL2NALv14 in PAML format [43]. These sequences were used along with a phylogeny tree in the CODEML module of the PAML4.9j package [44]. Site models (**M0**-one ratio, **M1**-nearly neutral, **M2a**-positive selection, **M3**-discrete, **M7**-beta, **M8**-beta, and ω > 1, **M8a**-beta and ω = 1) were used for these sequences and individual dN/dS ratio was also calculated for each branch. The hypothesis of different rates of evolution amongst different groups (*Leptocardii, Condricthyes, Coelacanthimorpha, Actinopteri, Amphibia, Cryptodira, Crocodylia, Bifurcata, Aves, Mammals*) were also tested. The phylogeny used considered branch lengths obtained from the Maximum Likelihood. Also, species from the *Leptocardii* group was included in both TRIF and TRAM phylogeny as an outgroup to root the phylogeny tree.

The parameters used for codeml run of site model were as shown in the table in Additional File 12.

The site model was performed for both protein family (TRAM and TRIF) using three models; **M3** versus **M0**, **M1** versus **M2a,** and **M7** versus **M8** for comparing positive selection. For identifying the potential amino acid residues that would have been under selection, we performed a Likelihood ratio test calculation for pairwise comparison of codon models using the Bayes empirical Bayes (BEB) method. The residues with probability above 0.95 and above were documented. Additionally, values for BEB or posterior probabilities of model **M8** for 11 site classes (k = 11) along the sequence of protein was plotted. The values from 11 site classes were grouped into two categories as ω > 1 and ω < 1. Also, the mean probabilities for each site were examined.

## Supplementary Information

**Additional file 1: Figs. S1–S6.**

**Additional file 2:** Amino acid sequence of TLR family proteins from 10 different orthologues.

**Additional file 3:** A maximum likelihood tree for TLR family proteins from 10 different orthologues.

**Additional file 4:** A detailed phylogeny with amino acid distance, domain architecture and motif conservation of TRIF orthologues.

**Additional file 5:** A detailed phylogeny with amino acid distance, domain architecture and motif conservation of TRAM orthologues.

**Additional file 6:** Protein Sequence ID’s and respective organism name of TRIF orthologues.

**Additional file 7:** Multiple Sequence Alignment of TRIF orthologues in FASTA format.

**Additional file 8:** Protein Sequence ID’s and respective organism name of TRAM orthologues.

**Additional file 9:** Multiple Sequence Alignment of TRAM orthologues in FASTA format.

**Additional file 10:** Protein Sequence ID’s and respective organism name of TAG orthologues.

**Additional file 11:** Multiple Sequence Alignment of TAG orthologues in FASTA format.

**Additional file 12:** The parameters and values used for codeml run of site model.

**Additional file 13:** Table showing domain architecture of TRIF and TRAM orthologue along with domain boundaries and e-value.

**Additional file 14:** List of hits obtained from genome wide search of TRIF orthologues using CS BLAST after filtering.

**Additional file 15:** List of hits obtained from genome wide search of TRAM and TAG orthologues using CS BLAST after filtering.

## Data Availability

The authors declare that [the/all other] data supporting the findings of this study are available within the article [and its supplementary information files].
