# Supplementary material for: A genome-wide search of Toll/Interleukin-1 receptor (TIR) domain-containing adapter molecule (TICAM) and their evolutionary divergence from other TIR domain containing proteins
Source: Biol Direct. 2022 Sep 2;17:24. doi: 10.1186/s13062-022-00335-9 (PMC9440496; doi:10.1186/s13062-022-00335-9)
Supplement: Supplementary file 1 — Additional file 1: Figs. S1–S6. [file 13062_2022_335_MOESM1_ESM.pdf]

Additional file 1

The position of conserved motifs for these sequences along with a gapped local alignment of motifs for these proteins is shown in Additional file 1: Fig S1-A and Fig S2. Motif logo, respective e-value, the number of sequences with motif sites, and length of motifs are shown in Additional file 1: Fig S1-B. These predicted four motifs correspond to previously reported Box1 (FDAFISY), Box2 (GYKLC-RD-PG), which are involved in the binding of proteins involved in signaling, and Box3 (a conserved W surrounded by basic residues) which is mainly involved in directing localization of receptor, through interactions with cytoskeletal elements [3]. But apart from that, an additional motif represented as common-4 was found to be present in all the TIR-containing proteins. Within these human proteins, Common-4 was the only motif found in adaptor TRAM/TICAM2 and TRIF/TICAM1. On tracing this sequence motif on TRAM-TIR structure from PDB (PDB id:2M1W) we found this region to be on the beta-sheet connecting the BB loop and αC helix [11]. A cartoon representation of the same has been shown in Additional file 1: Fig S1-C

Figure S1

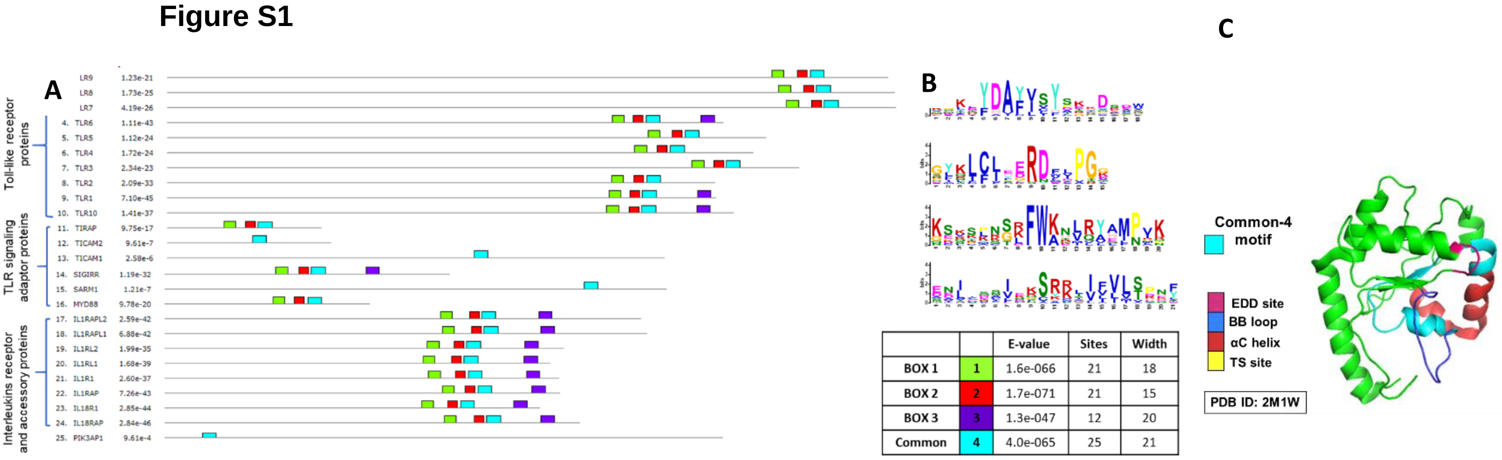

Figure S1: **A-** Image shows the conserved motifs positions present in Human TIR containing proteins. **B-** Motif logo, respective e-value, the number of sequences with motif sites, and length of motifs. **C-** Cartoon representation of common-4 motif on TRAM-TIR.

Figure S2

NAME	START	SITES	END	STRAND	MARGINAL SCORE
TLR9	870	YDAFVV.FDKT.........QSAV.ADWVYNELRGQLEECRGR..WALRLCLEERDWLP.GK	917	+	68.7
TLR8	880	YDAYISYDTK..........DASV.TDWVINELRYHLEESRD...KNVLLCLEERDWDP.GL	926	+	65.3
TLR7	891	YDAFIVYDTK..........DPAV.TEWVLAELVAKLEDPRE...KHFNLCLEERDWLP.GQ	937	+	65.4
TLR6	642	FHAFIS.............YSEHDSAWVKSELVPYLEK......EDIQICLHERNFVP.GK	682	+	79.4
TLR5	693	YDAYLC.............FSSKDFTWVQNALLKHL.DTQYSDQNRFNLCFEERDFVP.GE	738	+	49.4
TLR4	674	YDAFVI.............YSSQDEDWVRNELVKNLEEGV....PPFQLCLHYRDFIP.GV	716	+	76.6
TLR3	756	YAAYII.............HAYKDKDWVWEHFSSM.EKED....QSLKFCLEERDFEA.GV	797	+	29.9
TLR2	641	YDAFVS.............YSERDAYWVENLMVQELENFN....PPFKLCLHKRDFIP.GK	683	+	73.7
TLR1	637	FHAFIS.............YSGHDSFWVKNELLPNLEK......EGMQICLHERNFVP.GK	677	+	82.8
TLR10	634	FHAFIS.............YSEHDSLWVKNELIPNLEKED....GSILICLYESYFDP.GK	676	+	57.8
TIRAP	86	YDVCVC.............HSEEDLV.AAQDLVSYLEGST....ASLRCFLQLRDATP.GG	127	+	22.3
SIGIRR	165	YDAYVSYS............DCPEDRKFVNFILKPQLERR.....RGYKLFLDDRDLLPRAE	209	+	57.9
MYD88	161	FDAFIC.............YCPSDIQFV.QEMIRQLEQTN....YRLKLCVSDRDVLP.GT	202	+	56.2
IL1RAPL2	402	YDAYLSYTKVDQDTLDC...DNPEEEQFALEVLPDVLEKH.....YGYKLFIPERDLIPSGT	455	+	79.8
IL1RAPL1	405	YDAYLSYTKVDPDQWNQ...ETGEEERFALEILPDMLEKH.....YGYKLFIPDRDLIPTGT	458	+	73.9
IL1RL2	383	YDAYVLYPKPHKESQ.....RPAVDAL.VLNILPEVLERQ.....CGYKLFIFGRDEFP.GQ	432	+	69.1
IL1RL1	377	YDAYVVYPRNYKSSTDG...ASRVEHF.VHQILPDVLENK.....CGYTLCIYGRDMLP.GE	428	+	73.8
IL1R1	385	YDAYILYPKTVGEGS.....TSDCDIF.VFKVLPEVLEKQ.....CGYKLFIYGRDDYV.GE	434	+	54.8
IL1RAP	405	YDIYVSYA............RNAEEEEFVLLTLRGVLENE.....FGYKLCIFDRDSLP.GG	448	+	71.1
IL18R1	375	YDAFVSYLKECRP.......ENGEEHTFAVEILPRVLEKH.....FGYKLCIFERDVVP.GG	423	+	86.8
IL18RAP	408	FDAFVSYAKWSSFPSEATSSLSEE.HL.ALSLFPDVLENK.....YGYSLCLLERDVAP.GG	461	+	59.3

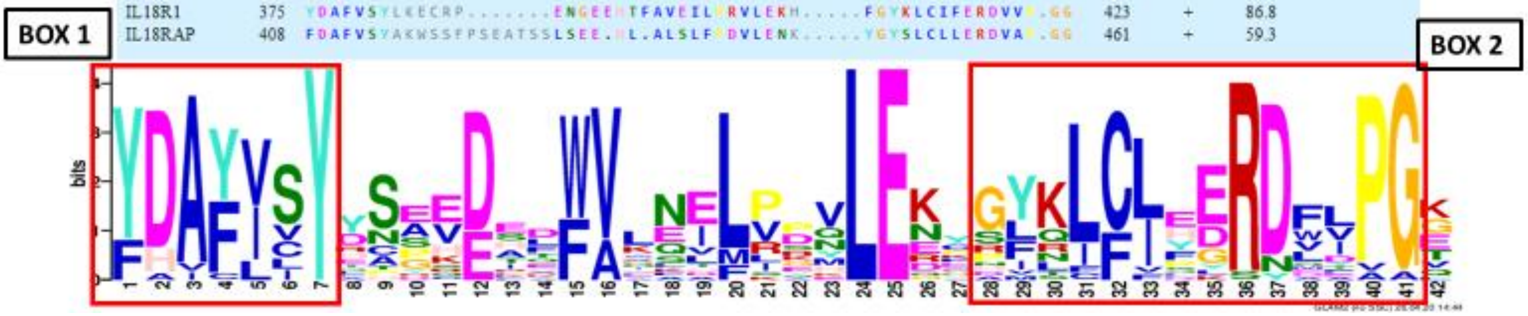

Figure S2: A gapped local alignment for human TIR containing proteins. Box 1 and Box 2 are highlighted in red.

Figure S3

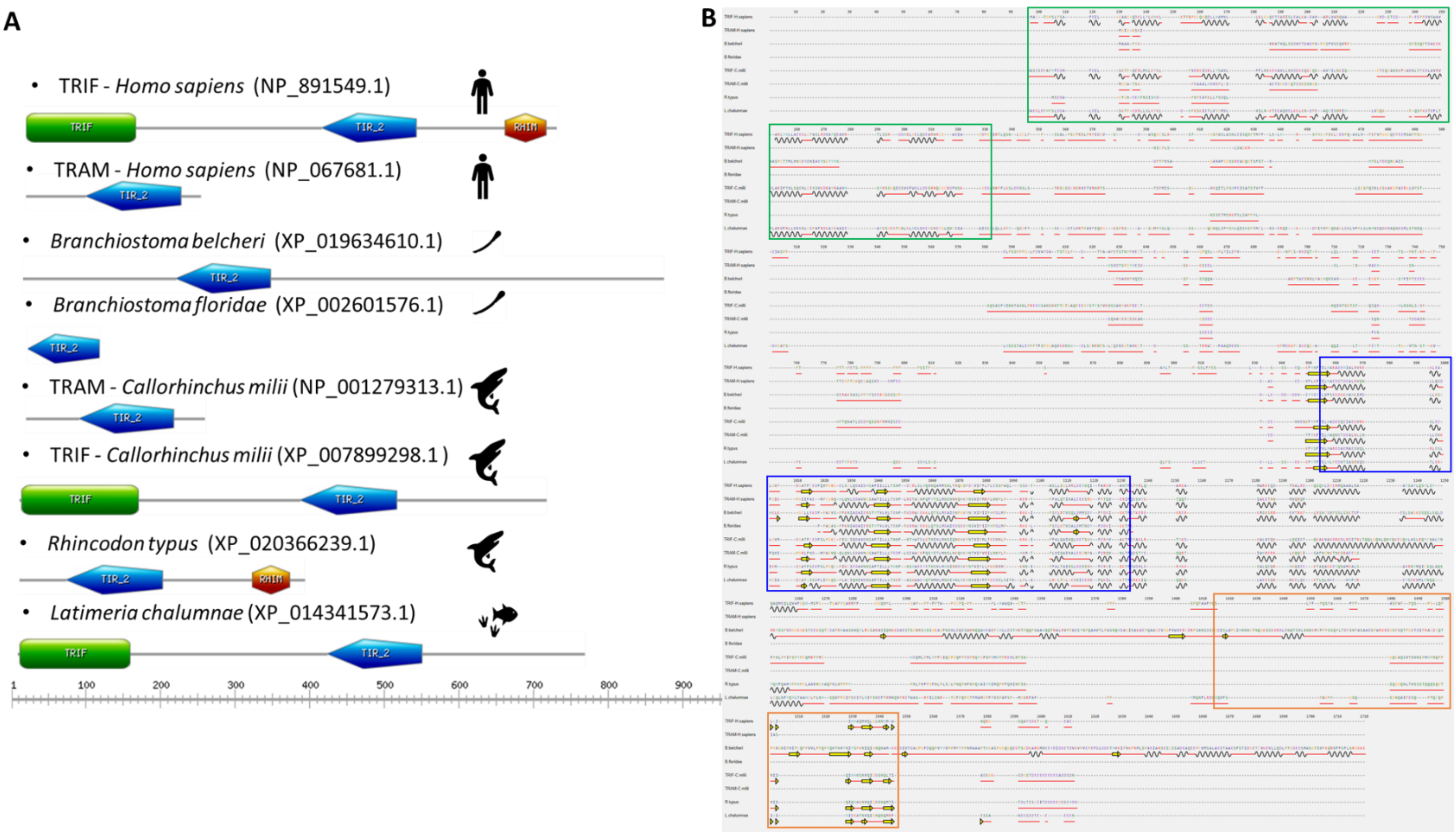

Figure S3: A. Some atypical sequences from the oldest ancestors. Its domain architecture and sequence ID. **B.** Secondary structure prediction for these sequences and alignment. TRIF-NTD (green), TIR_2 (blue), RHIM (orange) domains have been highlighted in the multiple sequence alignment.

Figure S4

CODEML site model for TRIF orthologues, to detect positively selected sites using BEB model.

Model	np	lnL	Model compared	2ΔlnL	LRT p values	Positive sites (BEB)
**M0**	2	-311196.7302	M0 vs M3	23316.1236	0	Not allowed
**M3**	6	-299538.6684		_		
**M1**	3	-297834.3405	M1 vs M2	11157.829	0	Not allowed
**M2**	5	-292255.426				3C,4T,20Q,33P,34R,35P,41D,50K,55T,65K,68A,71R,79G,80V,81D,83T,84E,111A,112S,126S,128R,129D,130D,131H,132R,134G,142N,148I,149A,151D,152P,153G,156R,157T,160S,161N,162L,164C,165L,166P,167P,168S,170A,171L,173S,174G,178L,180R,183D,184G,185V,186S,190Q,195R,217F,218L,219S,220L,222R,223S,224P,225H,228S,229K,232D,233D,234P,235Q,236A,238L,239V,240P,242P,243V,244P,245G,246G,247C,248Q,249E,250P,251E,254S,255W,256P,258S,261I,262A,263S,264P,266E,268P,269S,270S,271P,272P,273P,274G,275L,276P,277E,278V,279A,280P,281D,282A,283T,284S,285T,286G,287L,288P,289D,290T,291P,292A,293A,294P,295E,297S,298T,299N,300Y,301P,307G,308S,309A,310G,314L,315P,316L,318I,319L,320E,321P,323K,326C,332T,335Q,336L,340D,342T,343S,344P,352T,353P,354T,355T,356P,357E,358T,359S,363P,364P,366P,367P,368S,369S,371P,373S,374A,378P,379S,380S,381L,382F,383P,384S,385S,387E,389S,390S,392Q,508S,518Q,522R,525A,529K,534Q,535A,542K,546T,552Q,553S,555H,556L,558G,561M,562Q,563A,564A,565A,566L,567N,568A,570Y,571S,574L,575Q,576S,577Y,578L,579S,580Y,581Q,582A,583Q,584M,585E,586Q,588Q,589V,590A,592G,593S,594H,595M,596S,597F,598G,599T,600G,601A,603Y,604G,605A,606R,608P,609F,610G,611G,615L,617A,619P,621F,623T,624W,626G,627C,628P,629Q,633L,634H,635A,636W,637Q,638A,639G,640T,642P,645S,646P,647Q,648P,650A,652P,656P,661P,662A,663F,665T,666A,667S,668P,670P,673S,701S,702Q,703A,705E,708T,711A
**M7**	3	-289423.4733	M7 vs M8	5389.6354	0	Not allowed
**M8**	5	-286728.6556				3C,4T,34R,80V,83T,84E,111A,126S,128R,129D,131H,132R,149A,156R,162L,164C,166P,167P,168S,170A,171L,173S,174G,178L,180R,183D,184G,185V,186S,190Q,218L,220L,222R,223S,225H,229K,232D,233D,234P,235Q,236A,238L,239V,240P,242P,243V,244P,245G,246G,247C,248Q,249E,250P,251E,254S,255W,256P,258S,263S,266E,268P,269S,270S,271P,272P,273P,274G,275L,276P,277E,278V,279A,280P,281D,282A,283T,284S,285T,286G,287L,288P,289D,290T,291P,292A,293A,294P,295E,297S,298T,299N,300Y,301P,307G,308S,309A,310G,314L,315P,316L,318I,319L,320E,321P,323K,326C,332T,335Q,336L,340D,342T,343S,348P,349C,352T,353P,354T,355T,356P,357E,363P,364P,367P,368S,369S,371P,373S,374A,378P,379S,380S,382F,383P,384S,385S,389S,390S,508S,546T,552Q,555H,558G,562Q,563A,564A,565A,567N,571S,574L,575Q,576S,577Y,578L,579S,580Y,581Q,582A,583Q,584M,585E,586Q,588Q,589V,590A,592G,596S,597F,598G,599T,601A,603Y,604G,605A,606R,608P,609F,610G,619P,621F,623T,624W,626G,627C,628P,629Q,633L,634H,636W,637Q,638A,639G,640T,642P,645S,646P,647Q,648P,650A,661P,665T,666A,667S,670P,673S,701S,702Q,703A,708T,711A

Model	np	lnL	Model compared	2ΔlnL	LRT p values	Positive sites (BEB)
M0	2	-311197	M0 vs M3	23316.12	0	Not allowed
M3	6	-299539				_
M1	3	-297834	M1 vs M2	11157.83	0	Not allowed
M2	5	-292255				3C,4T,20Q,33P,34R,35P,41D,50K,55T,65K,68A,71R,79G,80V,81D,83T,84E,111A,112S,126S,128R,129D,130D,131H,132R,134G,142N,148I,149A,151D,152P,153G,156R,157T,160S,161N,162L,164C,165L,166P,167P,168S,170A,171L,173S,174G,178L,180R,183D,184G,185V,186S,190Q,195R,217F,218L,219S,220L,222R,223S,224P,225H,228S,229K,232D,233D,234P,235Q,236A,238L,239V,240P,242P,243V,244P,245G,246G,247C,248Q,249E,250P,251E,254S,255W,256P,258S,261I,262A,263S,264P,266E,268P,269S,270S,271P,272P,273P,274G,275L,276P,277E,278V,279A,280P,281D,282A,283T,284S,285T,286G,287L,288P,289D,290T,291P,292A,293A,294P,295E,297S,298T,299N,300Y,301P,307G,308S,309A,310G,314L,315P,316L,318I,319L,320E,321P,323K,326C,332T,335Q,336L,340D,342T,343S,344P,352T,353P,354T,355T,356P,357E,358T,359S,363P,364P,366P,367P,368S,369S,371P,373S,374A,378P,379S,380S,381L,382F,383P,384S,385S,387E,389S,390S,392Q,508S,518Q,522R,525A,529K,534Q,535A,542K,546T,552Q,553S,555H,556L,558G,561M,562Q,563A,564A,565A,566L,567N,568A,570Y,571S,574L,575Q,5[illegible] 636W,637Q,638A,639G,640T,642P,645S,646P,647Q,648P,650A,652P,656P,661P,662A,663F,665T,666A,667S,668P,670P,673S,701S,702Q,703A,705E,708T,711A
M7	3	-289423	M7 vs M8	5389.635	0	Not allowed
M8	5	-286729				3C,4T,34R,80V,83T,84E,111A,126S,128R,129D,131H,132R,149A,156R,162L,164C,166P,167P,168S,170A,171L,173S,174G,178L,180R,183D,184G,185V,186S,190Q,218L,220L,222R,223S,225H,229K,232D,233D,234P,235Q,236A,238L,239V,240P,242P,243V,244P,245G,246G,247C,248Q,249E,250P,251E,254S,255W,256P,258S,263S,266E,268P,269S,270S,271P,272P,273P,274G,275L,276P,277E,278V,279A,280P,281D,282A,283T,284S,285T,286G,287L,288P,289D,290T,291P,292A,293A,294P,295E,297S,298T,299N,300Y,301P,307G,308S,309A,310G,314L,315P,316L,318I,319L,320E,321P,323K,326C,332T,335Q,336L,340D,342T,343S,348P,349C,352T,353P,354T,355T,356P,357E,363P,364P,367P,368S,369S,371P,373S,374A,378P,379S,380S,382F,383P,384S,385S,389S,390S,508S,546T,552Q,555H,558G,562Q,563A,564A,565A,567N,571S,574L,575Q,576S,577Y,578L,579S,580Y,581Q,582A,583Q,584M,585E,586Q,588Q,589V,590A,592G,596S,597F,598G,599T,601A,603Y,604G,605A,606R,608P,609F,610G,619P,621F,623T,624W,626G,627C,628P,629Q,633L,634H,636W,637Q,638A,639G,640T,642P,645S,646P,647Q,648P,650A,661P,665T,666A,667S,670P,673S,701S,702Q,703A,708T,711A

np number of parameters; **lnL** log-likelihood value; **df** degree of freedom; **BEB model**; Bayes Empirical Bayes model.

Figure S5

CODEML site model for TRAM orthologues, to detect positively selected sites using BEB model.

Model	np	lnL	Model compared	2ΔlnL	LRT p values	Positive sites (BEB)

M0	2	-39457.5	M0 vs M3	6127.075	0	Not allowed
M3	6	-36394				_
M1	3	-36848.7	M1 vs M2	636.8226	0	Not allowed
M2	5	-36530.3				4G,8I,10S,14S,15L,16S,17W,25T,27P,29Y,30H, 32S,33D,34S,36K,37S,39D,40L,41S,43C,44N,45V, 47E,48H,49S,50N,51T,52T,54G,56T,59Q
M7	3	-36684.9	M7 vs M8	708.7464	0	Not allowed
M8	5	-36330.5				8I,14S,16S,17W,27P,29Y,30H,32S,33D,34S,36K, 37S,39D,40L

np number of parameters; **lnL** log-likelihood value; **df** degree of freedom; **BEB model**; Bayes Empirical Bayes model.

Figure S6

Domain annotation of *Crocodylus porosus* (HMM scan, inclusion e-value=0.048)

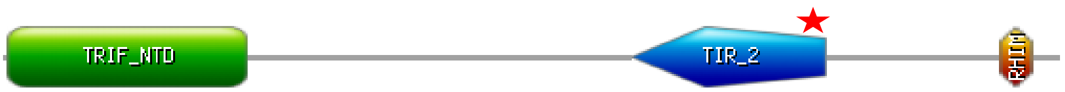

Length of protein = 692 amino acids

	Accession	Description	start	end	Inclusion e value
TRIF_NTD	PF17798.4	TRIF N-terminal domain	4	161	1.2e-39
TIR_2	PF13676.9	TIR domain	412	539	0.048 ★
RHIM	PF12721.10	RIP homotypic interaction motif	653	674	1.5e-06

Insignificant annotation ★
