# Supplementary material for: A genome-wide search of Toll/Interleukin-1 receptor (TIR) domain-containing adapter molecule (TICAM) and their evolutionary divergence from other TIR domain containing proteins
Source: Biol Direct. 2022 Sep 2;17:24. doi: 10.1186/s13062-022-00335-9 (PMC9440496; doi:10.1186/s13062-022-00335-9)

Additional File 3

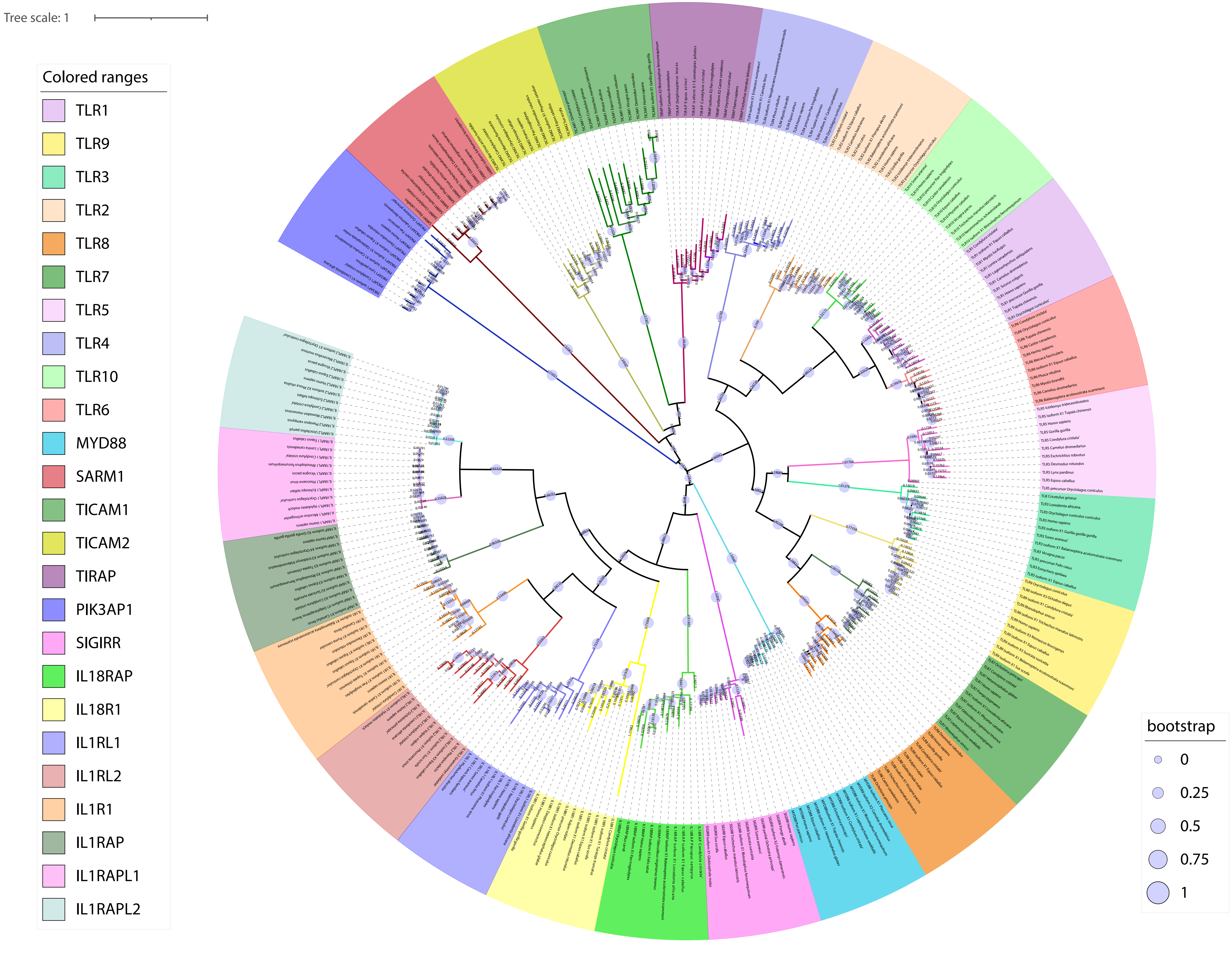

Additional File 3

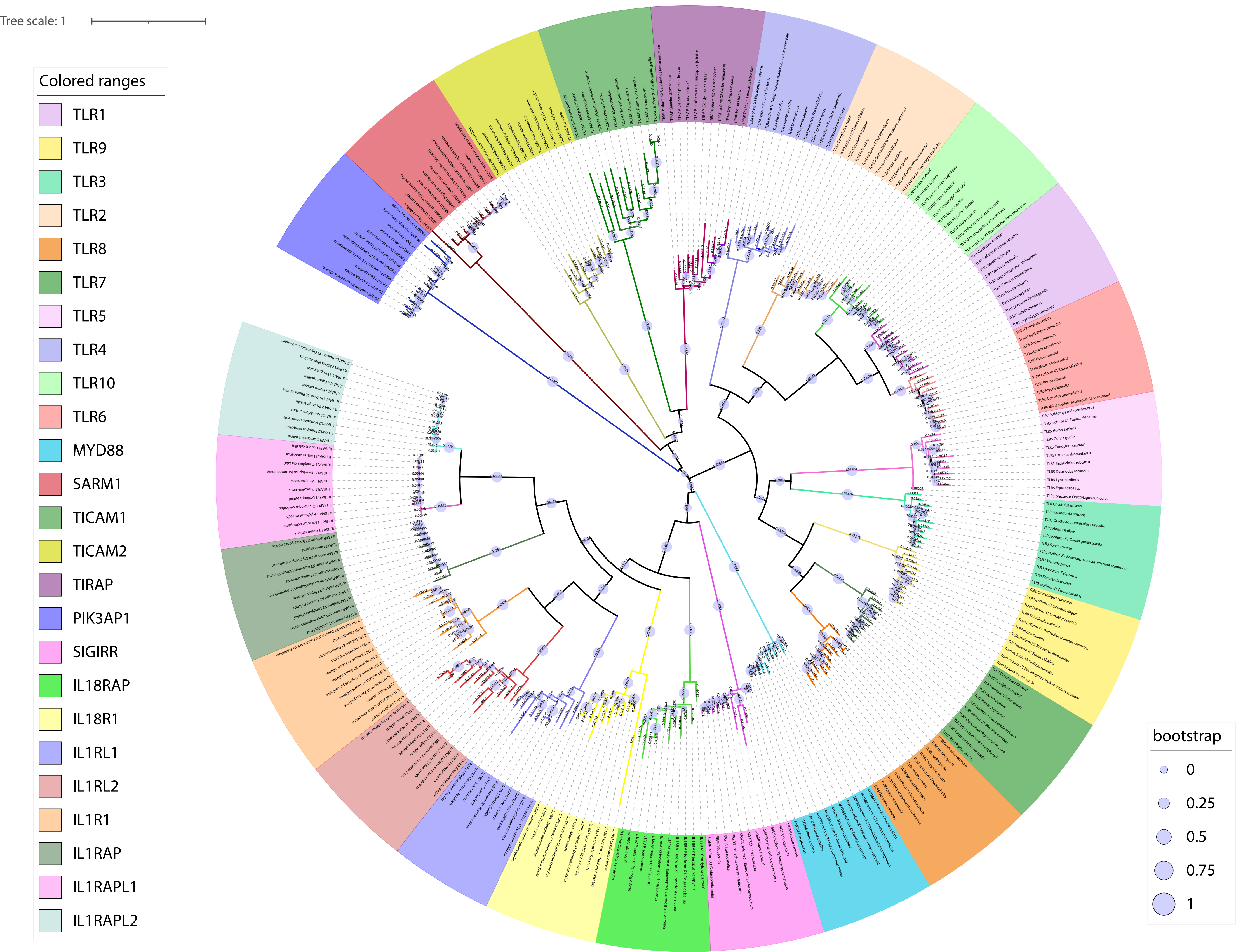

Supplement: Supplementary file 3 — Additional file 3: A maximum likelihood tree for TLR family proteins from 10 different orthologues. [file 13062_2022_335_MOESM3_ESM.pdf]
