# Supplementary figures and images for: A genome-wide search of Toll/Interleukin-1 receptor (TIR) domain-containing adapter molecule (TICAM) and their evolutionary divergence from other TIR domain containing proteins
Source: Biol Direct. 2022 Sep 2;17:24. doi: 10.1186/s13062-022-00335-9 (PMC9440496; doi:10.1186/s13062-022-00335-9)

Additional File 4

480 1500 1510

Tree scale: 1

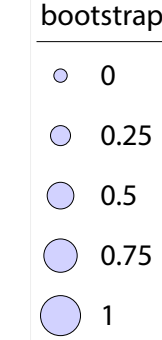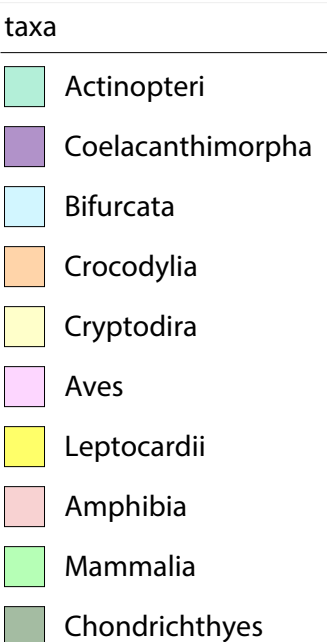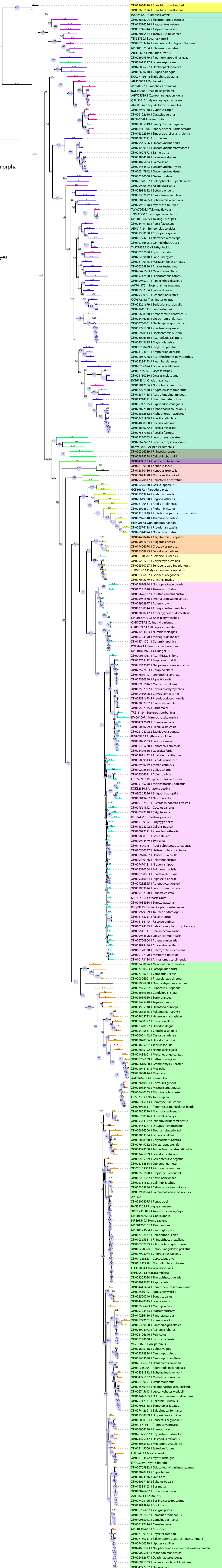

Supplement: Supplementary file 4 — Additional file 4: A detailed phylogeny with amino acid distance, domain architecture and motif conservation of TRIF orthologues. [file 13062_2022_335_MOESM4_ESM.pdf]

## Additional file 5

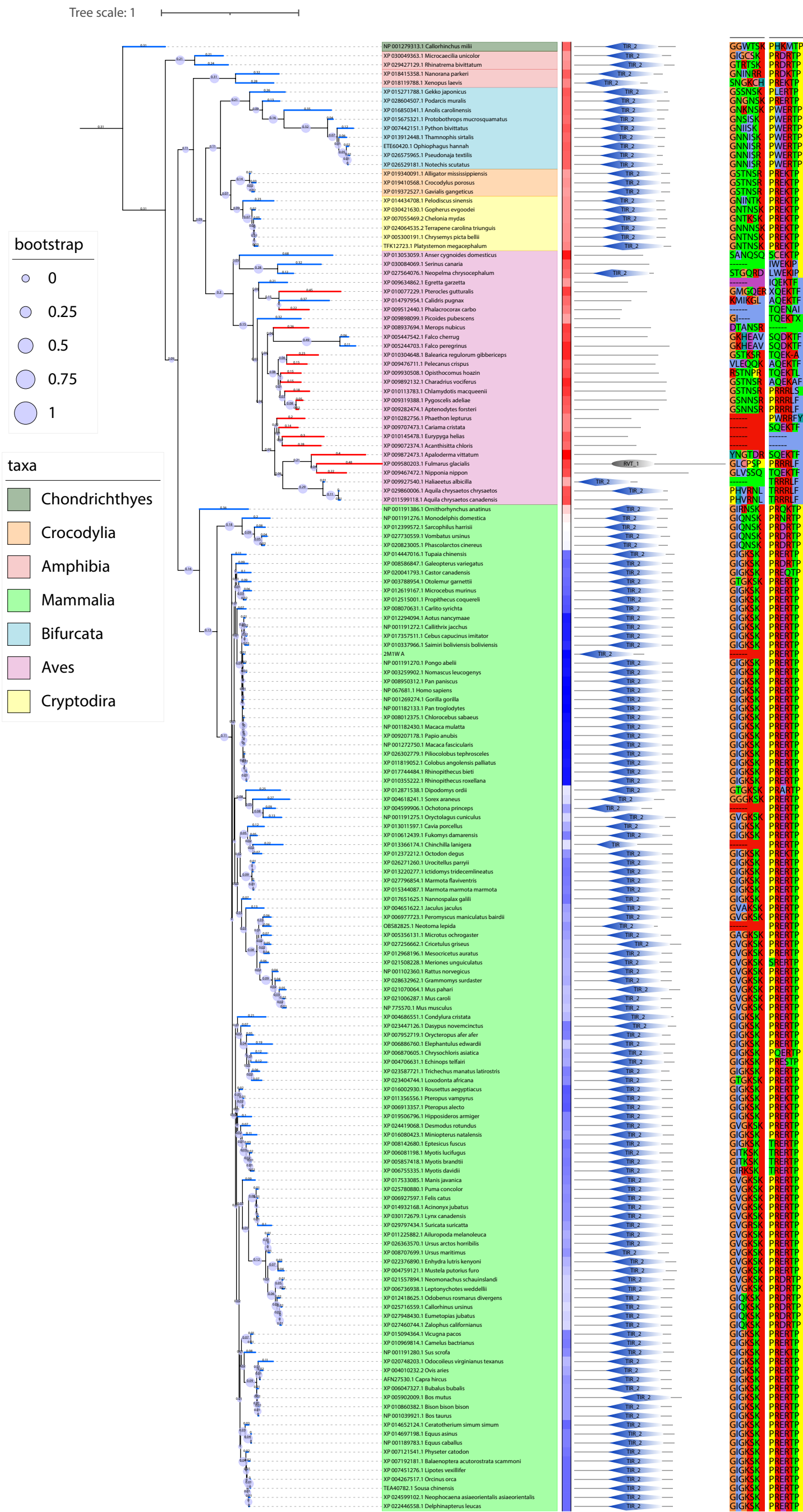

Supplement: Supplementary file 5 — Additional file 5: A detailed phylogeny with amino acid distance, domain architecture and motif conservation of TRAM orthologues. [file 13062_2022_335_MOESM5_ESM.pdf]
