# Supplementary material for: A genome-wide search of Toll/Interleukin-1 receptor (TIR) domain-containing adapter molecule (TICAM) and their evolutionary divergence from other TIR domain containing proteins
Source: Biol Direct. 2022 Sep 2;17:24. doi: 10.1186/s13062-022-00335-9 (PMC9440496; doi:10.1186/s13062-022-00335-9)
Supplement: Supplementary file 12 — Additional file 12: The parameters and values used for codeml run of site model. [file 13062_2022_335_MOESM12_ESM.pdf]

The parameters used for codeml run of site model

Parameters	Values
seqfile	x.PAML
treefile	x.nwk
outfile	mlc
noisy	3
verbose	0
runmode	0
seqtype	1
CodonFreq	2
aaRatefile	jones.dat
model	0
NSsites	0 1 2 3 7 8
icode	0
Mgene	0
fix_kappa	0
kappa	1.6
fix_omega	0
omega	.9
fix_alpha	1
alpha	0
ncatG	10
clock	0
getSE	0
RateAncestor	0
Small_Diff	.1e-6
method	1
fix_blength	2
