# Supplementary material for: A genome-wide search of Toll/Interleukin-1 receptor (TIR) domain-containing adapter molecule (TICAM) and their evolutionary divergence from other TIR domain containing proteins
Source: Biol Direct. 2022 Sep 2;17:24. doi: 10.1186/s13062-022-00335-9 (PMC9440496; doi:10.1186/s13062-022-00335-9)
Supplement: Supplementary file 13 — Additional file 13: Table showing domain architecture of TRIF and TRAM orthologue along with domain boundaries and e-value. [file 13062_2022_335_MOESM13_ESM.pdf]

TRIF orthologues

Node ID	domain	domain boundary	e-value	domain	domain boundary	e-value	domain	domain boundary	e-value
2M1X_A	TIR_2	12-140	8.20E-04		-			-	
AAH37048.2_Mus_musculus	TRIF_NTD	59-206	2.10E-39	TIR_2	457-588	1.40E-03	RHIM	668-756	7.20E-10
AAI51623.1_Bos_taurus	TRIF_NTD	1-148	6.50E-52	RHIM	691-744	1.10E-09		-	
ABH10662.1_Ictalurus_furcatus	TIR_2	34-165	4.60E-09		-			-	
ABH10822.1_Danio_rerio	TRIF_NTD	9-158	5.10E-51	TIR_2	322-452	5.60E-07		-	
AEX01719.1_Epinephelus_coioides	TRIF_NTD	6-156	9.00E-40	TIR_2	334-481	1.20E-03		-	
AGW25589.1_Ctenopharyngodon_idella	TRIF_NTD	6-155	1.30E-40	TIR_2	314-442	8.30E-07		-	
AMP81962.1_Squaliobarbus_curriculus	TRIF_NTD	6-155	4.10E-40	TIR_2	314-442	1.90E-06		-	
AWP04170.1_Scophthalmus_maximus	TRIF_NTD	6-162	5.00E-42	TIR_2	331-459	6.40E-08		-	
AYN78122.1_Pimephales_promelas	TRIF_NTD	7-162	3.90E-38	TIR_2	321-450	2.00E-05		-	
BAG55265.1_Pongo_pygmaeus	TRIF_NTD	1-148	1.20E-49	TIR_2	396-523	3.20E-03	RHIM	641-696	4.10E-12
EHH29504.1_Macaca_mulatta	TRIF_NTD	1-148	2.00E-49	TIR_2	396-524	5.20E-03	RHIM	641-696	1.80E-12
EHH59094.1_Macaca_fascicularis	TRIF_NTD	1-148	1.20E-49	TIR_2	404-532	8.30E-03	RHIM	649-704	1.80E-12
ELK37433.1_Myotis_davidii	TRIF_NTD	1-141	3.20E-43		-			-	
EPQ03069.1_Myotis_brandtii	TRIF_NTD	1-36	6.40E-05	RHIM	461-507	9.50E-11		-	
ETE59017.1_Ophiophagus_hannah	TRIF_NTD	15-170	1.80E-62	RHIM	576-629	1.90E-07		-	
GCF56572.1_Paroedura_picta	TRIF_NTD	4-157	7.80E-48	TIR_2	379-503	7.30E-04	RHIM	592-643	9.80E-05
KAA0711931.1_Triplophysa_tibetana	TRIF_NTD	13-166	4.30E-33	TIR_2	329-457	1.60E-07		-	
KFP48730.1_Cathartes_aura	TRIF_NTD	4-160	2.70E-54		-			-	
KFQ89713.1_Phoenicopterus_ruber_ruber	TRIF_NTD	4-160	1.20E-54	TIR_2	447-574	1.20E-03		-	
KFU84451.1_Chaetura_pelagica	TRIF_NTD	4-152	1.20E-20	TIR_2	444-572	1.40E-03		-	
KQK82830.1_Amazona_aestiva	TRIF_NTD	4-159	7.00E-47	TIR_2	437-564	4.20E-03	RHIM	629-679	4.30E-09
NP_001074975.1_Gallus_gallus	TRIF_NTD	4-157	2.20E-43	TIR_2	442-567	7.00E-06	RHIM	659-717	4.50E-11
NP_001106665.1_Takifugu_rubripes	TRIF_NTD	30-183	1.60E-33		-			-	
NP_001123604.1_Pan_troglodytes	TRIF_NTD	1-148	1.80E-50	TIR_2	398-525	6.20E-03	RHIM	643-698	2.30E-12
NP_001187154.1_Ictalurus_punctatus	TIR_2	249-379	1.50E-08		-			-	
NP_001266155.1_Pan_paniscus	TRIF_NTD	1-148	1.80E-50	TIR_2	396-523	6.20E-03	RHIM	641-696	2.30E-12
NP_001266518.1_Gorilla_gorilla	TRIF_NTD	1-148	1.80E-50	TIR_2	397-524	6.20E-03	RHIM	642-697	7.40E-13
NP_001297720.1_Anas_platyrhynchos	TRIF_NTD	4-160	8.10E-45	TIR_2	445-568	1.20E-05	RHIM	646-716	3.90E-11
NP_001302667.1_Sus_scrofa	TRIF_NTD	1-148	2.20E-52	TIR_2	393-524	1.80E-03	RHIM	647-698	7.00E-09
NP_891549.1_Homo_sapiens	TRIF_NTD	1-148	1.80E-50	TIR_2	397-524	6.20E-03	RHIM	642-697	7.40E-13
OBS82883.1_Neotoma_lepida	TRIF_NTD	1-148	1.30E-42	TIR_2	398-528	4.00E-04	RHIM	594-631	9.80E-08
OPJ77699.1_Patagioenas_fasciata_monilis	TIR_2	16-143	1.70E-06	RHIM	198-260	1.70E-06		-	
OXB58717.1_Callipepla_squamata	TRIF_NTD	5-157	1.50E-44	TIR_2	443-570	9.20E-05	RHIM	654-718	9.70E-10
OXB70737.1_Colinus_virginianus	TRIF_NTD	5-157	4.10E-44	TIR_2	437-564	5.20E-05	RHIM	644-712	1.30E-08
POI34423.1_Bambusicola_thoracicus	TRIF_NTD	4-157	8.10E-43	TIR_2	442-567	2.10E-04	RHIM	655-717	1.80E-10
PWA25120.1_Gambusia_affinis	TRIF_NTD	82-140	5.00E-09	TIR_2	215-345	1.70E-05		-	
QAU56515.1_Mylopharyngodon_piceus	TRIF_NTD	6-155	3.90E-40	TIR_2	314-442	8.30E-07		-	
QCV57275.1_Trachinotus_ovatus	TRIF_NTD	6-162	1.50E-40	TIR_2	338-466	4.00E-08		-	
RLV90980.1_Erythrura_gouldiae	TRIF_NTD	4-153	1.80E-45	TIR_2	409-536	5.50E-03	RHIM	598-653	3.50E-08
RMC01007.1_Hirundo_rustica_rustica	TIR_2	14-138	1.20E-04		-			-	
ROL54960.1_Anabarilius_grahami	TRIF_NTD	2-151	5.30E-40	TIR_2	310-438	1.70E-06		-	
RVE61834.1_Oryzias_javanicus	TRIF_NTD	6-154	8.50E-38	TIR_2	331-459	3.20E-03		-	

RXM93555.1_Acipenser_ruthenus	TRIF_NTD	4-157	5.10E-32	TIR_2	413-540	2.10E-05	RHIM	668-716	7.50E-03
RXN20798.1_Labeo_rohita	TRIF_NTD	9-158	4.70E-38	TIR_2	313-443	1.80E-06		-	
TEA33334.1_Sousa_chinensis	TRIF_NTD	1-148	8.40E-51	TIR_2	391-528	9.00E-03	RHIM	645-692	1.30E-10
TFK00140.1_Platysternon_megacephalum	TRIF_NTD	4-157	7.50E-48	TIR_2	393-520	1.20E-04	RHIM	616-673	4.30E-08
TKS79933.1_Collichthys_lucidus	TRIF_NTD	6-162	8.90E-45	TIR_2	349-477	5.80E-05		-	
TNM97751.1_Takifugu_bimaculatus	TRIF_NTD	30-183	8.80E-34		-			-	
TRZ11119.1_Zosterops_borbonicus	TRIF_NTD	4-145	3.70E-37	TIR_2	417-545	1.50E-04	RHIM	573-650	7.60E-06
TSK53730.1_Bagarius_yarrelli	TIR_2	245-376	2.20E-05		-			-	
TWW72608.1_Takifugu_flavidus	TRIF_NTD	6-159	8.10E-34		-			-	
VFV19469.1_Lynx_pardinus	TRIF_NTD	1-148	5.80E-50	RHIM	539-580	7.40E-11		-	
XP_002194292.2_Taeniopygia_guttata	TRIF_NTD	4-152	1.30E-43	TIR_2	423-551	4.40E-03	RHIM	616-670	1.50E-07
XP_002601576.1_Branchiostoma_floridae	TIR_2	1-96	2.30E-10		-			-	
XP_002761653.1_Callithrix_jacchus	TRIF_NTD	1-148	2.90E-49	TIR_2	394-522	9.20E-03	RHIM	624-682	3.80E-12
XP_003421709.1_Loxodonta_africana	TRIF_NTD	1-148	2.80E-43	RHIM	640-685	1.50E-09		-	
XP_003460977.1_Cavia_porcellus	TRIF_NTD	1-148	9.20E-44	RHIM	586-663	1.90E-03		-	
XP_003760653.1_Sarcophilus_harrisii	TRIF_NTD	1-152	3.90E-46	TIR_2	401-528	2.60E-03	RHIM	624-675	9.10E-09
XP_003788814.1_Otolemur_garnettii	TRIF_NTD	1-148	2.10E-50	RHIM	606-674	1.60E-08		-	
XP_003919632.2_Papio_anubis	TRIF_NTD	1-148	1.20E-49	TIR_2	398-528	7.90E-03	RHIM	629-698	3.60E-12
XP_003938874.1_Saimiri_boliviensis_boliviensis	TRIF_NTD	1-148	5.50E-49	RHIM	626-683	4.30E-11		-	
XP_004023546.3_Ovis_aries	TRIF_NTD	1-148	3.60E-52	RHIM	697-750	6.50E-10		-	
XP_004277282.1_Orcinus_orca	TRIF_NTD	1-148	1.70E-51	TIR_2	391-528	9.00E-03	RHIM	646-692	1.60E-10
XP_004312297.2_Tursiops_truncatus	TRIF_NTD	1-148	1.30E-51	TIR_2	391-520	7.70E-03		-	
XP_004378500.1_Trichechus_manatus_latirostris	TRIF_NTD	3-150	2.40E-41	RHIM	644-688	8.60E-10		-	
XP_004441454.1_Ceratotherium_simum_simum	TRIF_NTD	1-148	8.10E-48	TIR_2	382-519	7.80E-03	RHIM	632-687	4.70E-11
XP_004464287.1_Dasypus_novemcinctus	TRIF_NTD	3-148	4.20E-41	RHIM	569-657	3.10E-10		-	
XP_004542615.2_Maylandia_zebra	TRIF_NTD	6-164	5.30E-39	TIR_2	337-467	2.00E-05		-	
XP_004595948.1_Ochotona_princeps	TRIF_NTD	1-148	3.10E-46	RHIM	585-641	3.60E-10		-	
XP_004619550.1_Sorex_araneus	TRIF_NTD	1-149	2.20E-39	RHIM	632-674	4.60E-09		-	
XP_004655027.1_Jaculus_jaculus	TRIF_NTD	1-148	1.10E-43	TIR_2	397-534	9.60E-04	RHIM	623-691	2.30E-09
XP_004689386.1_Condylura_cristata	TRIF_NTD	1-148	1.10E-41	TIR_2	381-517	8.40E-04	RHIM	596-663	3.90E-10
XP_004771537.1_Mustela_putorius_furo	TRIF_NTD	3-150	4.30E-49	RHIM	643-708	6.90E-10		-	
XP_004865772.1_Heterocephalus_glaber	TRIF_NTD	1-148	1.10E-40	TIR_2	390-520	1.80E-04	RHIM	624-680	4.00E-08
XP_005060299.1_Ficedula_albicollis	TRIF_NTD	4-154	3.30E-44	TIR_2	431-557	3.80E-03	RHIM	613-660	5.40E-08
XP_005088478.2_Mesocricetus_auratus	TRIF_NTD	69-216	8.80E-41	TIR_2	466-597	1.70E-03	RHIM	707-774	4.00E-09
XP_005155258.1_Melopsittacus_undulatus	TRIF_NTD	4-160	5.20E-46	TIR_2	437-563	2.70E-03	RHIM	626-677	3.60E-09
XP_005281227.1_Chrysemys_picta_bellii	TRIF_NTD	4-157	4.40E-51	TIR_2	406-534	3.10E-03	RHIM	628-685	5.40E-08
XP_005343119.2_Ictidomys_tridecemlineatus	TRIF_NTD	1-148	7.20E-49	TIR_2	390-519	3.10E-03		-	
XP_005405837.1_Chinchilla_lanigera	TRIF_NTD	1-148	1.00E-40	TIR_2	391-522	1.40E-03	RHIM	612-692	1.20E-04
XP_005429516.1_Geospiza_fortis	TRIF_NTD	4-152	2.40E-44	TIR_2	425-551	9.20E-04	RHIM	618-672	4.10E-08
XP_005476265.1_Oreochromis_niloticus	TRIF_NTD	6-163	5.40E-40	TIR_2	337-467	3.90E-05		-	
XP_005495270.1_Zonotrichia_albicollis	TRIF_NTD	4-154	8.40E-45	TIR_2	426-555	1.40E-03	RHIM	635-682	5.30E-08
XP_005504021.1_Columba_livia	TIR_2	339-465	6.30E-05	RHIM	521-583	3.40E-08		-	
XP_005531673.2_Pseudopodoces_humilis	TRIF_NTD	30-175	1.60E-42	TIR_2	446-567	3.80E-03	RHIM	623-692	2.60E-08
XP_005633080.1_Canis_lupus_familiaris	TRIF_NTD	1-148	1.40E-48	RHIM	692-736	2.60E-10		-	
XP_005721268.1_Pundamilia_nyererei	TRIF_NTD	6-164	5.70E-39	TIR_2	335-465	2.00E-05		-	
XP_005812562.1_Xiphophorus_maculatus	TRIF_NTD	6-159	1.70E-49	TIR_2	344-473	6.30E-06		-	
XP_005920513.1_Haplochromis_burtoni	TRIF_NTD	6-164	7.60E-39	TIR_2	337-467	2.00E-05		-	

XP_006067185.2_Bubalus_bubalis	TRIF_NTD	1-148	7.40E-53	RHIM	705-758	1.10E-09		-	
XP_006104892.1_Myotis_lucifugus	TRIF_NTD	1-149	8.50E-51	RHIM	587-672	5.30E-09		-	
XP_006110580.2_Pelodiscus_sinensis	TRIF_NTD	1-152	1.00E-42	TIR_2	387-512	1.90E-04	RHIM	606-664	6.00E-08
XP_006177626.1_Camelus_ferus	TRIF_NTD	1-148	8.60E-49	TIR_2	391-527	1.60E-03	RHIM	647-708	1.80E-10
XP_006206437.1_Vicugna_pacos	TRIF_NTD	1-148	1.40E-48	TIR_2	391-527	2.80E-03	RHIM	651-708	1.30E-10
XP_006745643.1_Leptonychotes_weddellii	TRIF_NTD	1-148	2.30E-49	RHIM	584-670	2.00E-10		-	
XP_006786661.1_Neolamprologus_brichardi	TRIF_NTD	6-164	7.60E-39	TIR_2	339-469	1.20E-05		-	
XP_006868978.1_Chrysochloris_asiatica	TRIF_NTD	1-148	1.50E-42	RHIM	619-676	3.50E-10		-	
XP_006896500.1_Elephantulus_edwardii	TRIF_NTD	1-148	1.90E-40	TIR_2	425-553	2.40E-03	RHIM	667-726	1.60E-10
XP_006904180.1_Pteropus_alecto	TRIF_NTD	1-149	1.20E-54	RHIM	632-679	1.40E-11		-	
XP_006982311.1_Peromyscus_maniculatus_bairdii	TRIF_NTD	1-148	3.90E-41	TIR_2	401-530	1.10E-03	RHIM	613-711	5.60E-07
XP_007072279.1_Chelonia_mydas	TRIF_NTD	4-157	8.30E-51	TIR_2	409-536	5.90E-05	RHIM	631-688	2.00E-08
XP_007109377.1_Physeter_catodon	TRIF_NTD	1-152	4.10E-54	RHIM	668-717	1.40E-09		-	
XP_007169217.1_Balaenoptera_acutorostrata_scammoni	TRIF_NTD	1-148	1.40E-52	TIR_2	391-521	4.40E-03	RHIM	643-690	9.90E-09
XP_007246244.2_Astyanax_mexicanus	TIR_2	355-480	1.10E-03		-			-	
XP_007460599.1_Lipotes_vexillifer	TRIF_NTD	1-148	4.90E-53	TIR_2	391-521	9.00E-03	RHIM	640-686	3.20E-10
XP_007488996.1_Monodelphis_domestica	TRIF_NTD	1-153	1.40E-59	TIR_2	412-539	3.10E-04	RHIM	637-686	2.80E-08
XP_007525002.2_Erinaceus_europaeus	TRIF_NTD	31-179	2.50E-30	RHIM	527-620	7.40E-05		-	
XP_007567988.1_Poecilia_formosa	TRIF_NTD	6-159	2.30E-54	TIR_2	346-475	1.00E-05		-	
XP_007640604.1_Cricetulus_griseus	TRIF_NTD	1-148	8.90E-39	TIR_2	400-521	1.90E-03	RHIM	635-704	2.10E-10
XP_007899298.1_Callorhinchus_milii	TRIF_NTD	4-161	6.60E-28	TIR_2	377-506	5.30E-07		-	
XP_007949252.1_Orycteropus_afer_afer	TRIF_NTD	1-148	2.40E-45	TIR_2	397-528	3.60E-03	RHIM	630-681	2.00E-11
XP_007993074.1_Chlorocebus_sabaeus	TRIF_NTD	1-148	5.40E-50	TIR_2	396-524	8.10E-03	RHIM	627-696	5.80E-12
XP_008120431.1_Anolis_carolinensis	TRIF_NTD	4-160	1.80E-64	TIR_2	479-610	3.40E-03	RHIM	663-709	2.80E-05
XP_008149009.1_Eptesicus_fuscus	TRIF_NTD	1-149	3.30E-49	RHIM	627-673	2.20E-12		-	
XP_008280479.1_Stegastes_partitus	TRIF_NTD	6-163	1.80E-42	TIR_2	354-511	1.60E-04		-	
XP_008332072.1_Cynoglossus_semilaevis	TRIF_NTD	14-170	1.10E-36	TIR_2	343-471	8.10E-03		-	
XP_008431969.1_Poecilia_reticulata	TRIF_NTD	6-159	3.40E-52	TIR_2	345-473	1.20E-04		-	
XP_008510112.1_Equus_przewalskii	TRIF_NTD	2-77	1.30E-23	TIR_2	159-294	5.30E-04		-	
XP_008585959.1_Galeopterus_variegatus	TRIF_NTD	1-148	3.80E-49	TIR_2	396-526	4.60E-03	RHIM	641-690	2.30E-11
XP_008709651.1_Ursus_maritimus	TRIF_NTD	1-148	2.20E-48	TIR_2	423-577	2.80E-03		-	
XP_008756139.2_Rattus_norvegicus	TRIF_NTD	52-199	5.60E-38	TIR_2	429-550	3.40E-03	RHIM	642-706	1.00E-10
XP_008835192.1_Nannospalax_galili	TRIF_NTD	1-148	3.40E-42	TIR_2	395-523	8.10E-03	RHIM	622-684	2.20E-09
XP_008931616.3_Manacus_vitellinus	TRIF_NTD	4-150	1.00E-48	TIR_2	425-548	1.60E-04		-	
XP_008946049.1_Merops_nubicus	TRIF_NTD	4-160	3.70E-51	TIR_2	450-576	1.10E-03	RHIM	616-703	1.50E-03
XP_009081957.1_Acanthisitta_chloris	TRIF_NTD	4-159	1.30E-47	TIR_2	419-545	3.00E-04	RHIM	576-652	2.10E-04
XP_009095163.2_Serinus_canaria	TRIF_NTD	4-150	2.40E-45	TIR_2	423-550	8.10E-04	RHIM	616-668	2.70E-08
XP_009282325.1_Aptenodytes_forsteri	TRIF_NTD	4-160	1.10E-55	TIR_2	453-573	1.00E-03	RHIM	663-713	1.00E-08
XP_009316684.1_Pygoscelis_adeliae	TRIF_NTD	4-160	4.00E-57	TIR_2	453-571	1.90E-03	RHIM	633-713	6.70E-06
XP_009470181.1_Nipponia_nippon	TRIF_NTD	4-160	1.50E-54	TIR_2	451-576	3.50E-04	RHIM	649-706	3.90E-08
XP_009480176.1_Pelecanus_crispus	TRIF_NTD	4-160	3.90E-57	TIR_2	439-565	9.30E-03	RHIM	642-699	4.60E-07
XP_009511631.1_Phalacrocorax_carbo	TRIF_NTD	4-160	1.20E-54	TIR_2	449-574	1.80E-04	RHIM	651-708	1.70E-09
XP_009561132.1_Cuculus_canorus	TRIF_NTD	4-159	1.60E-50	TIR_2	450-577	6.80E-04	RHIM	653-697	6.60E-07
XP_009570504.1_Fulmarus_glacialis	TRIF_NTD	4-159	3.00E-54	TIR_2	450-577	2.10E-03	RHIM	625-711	5.90E-07
XP_009634984.1_Egretta_garzetta	TRIF_NTD	4-160	1.20E-54	TIR_2	451-577	2.10E-03	RHIM	649-706	2.40E-07
XP_009676037.1_Struthio_camelus_australis	TRIF_NTD	4-160	1.60E-52	TIR_2	399-526	7.30E-03	RHIM	580-674	2.10E-08

XP_009707398.1_Cariama_cristata	TRIF_NTD	4-160	9.80E-56	TIR_2	451-577	5.10E-04	RHIM	626-711	2.90E-09
XP_009808101.1_Gavia_stellata	TRIF_NTD	4-160	1.10E-54	TIR_2	442-569	2.10E-03	RHIM	645-702	2.60E-08
XP_009871442.1_Apaloderma_vittatum	TRIF_NTD	4-161	1.20E-46	TIR_2	457-584	1.60E-03	RHIM	681-713	3.40E-04
XP_009893486.1_Charadrius_vociferus	TRIF_NTD	4-159	2.40E-51	TIR_2	452-571	3.20E-03	RHIM	628-712	3.80E-04
XP_009899013.1_Picoides_pubescens	TRIF_NTD	4-154	4.00E-46	TIR_2	445-569	5.30E-04	RHIM	629-702	3.70E-06
XP_009920467.1_Haliaeetus_albicilla	TRIF_NTD	4-160	8.60E-54	TIR_2	449-575	3.00E-03	RHIM	652-709	9.40E-09
XP_009934696.1_Opisthocomus_hoazin	TRIF_NTD	4-159	3.60E-52	TIR_2	451-576	1.60E-03	RHIM	627-711	6.30E-07
XP_009959824.1_Leptosomus_discolor	TRIF_NTD	4-160	1.70E-52	TIR_2	451-577	2.10E-03	RHIM	661-711	3.30E-08
XP_009974479.1_Tyto_alba	TRIF_NTD	4-167	5.40E-46	TIR_2	456-582	3.50E-04	RHIM	642-716	1.60E-07
XP_009979399.1_Tauraco_erythrolophus	TRIF_NTD	4-160	4.60E-53	TIR_2	449-575	4.50E-03	RHIM	652-702	2.00E-07
XP_010019457.1_Nestor_notabilis	TRIF_NTD	4-160	3.30E-48	RHIM	630-683	5.20E-09		-	
XP_010072321.1_Pterocles_gutturalis	TRIF_NTD	4-157	4.00E-49	TIR_2	444-568	3.30E-03	RHIM	601-654	1.10E-07
XP_010120978.1_Chlamydotis_macqueenii	TRIF_NTD	4-160	6.50E-57	TIR_2	451-577	2.00E-03	RHIM	655-710	6.90E-09
XP_010137501.1_Buceros_rhinoceros_silvestris	TRIF_NTD	4-160	2.40E-54	TIR_2	436-563	2.50E-03	RHIM	652-695	8.90E-08
XP_010159112.1_Eurypyga_helias	TRIF_NTD	4-153	8.90E-50	TIR_2	424-550	2.10E-04	RHIM	613-669	3.30E-09
XP_010173154.1_Antrostomus_carolinensis	TRIF_NTD	4-150	1.40E-45	TIR_2	441-567	2.10E-04	RHIM	615-701	1.30E-07
XP_010177190.1_Mesitornis_unicolor	TRIF_NTD	4-158	1.70E-51	TIR_2	444-571	2.50E-03	RHIM	601-704	8.70E-06
XP_010202854.1_Colius_striatus	TIR_2	196-320	6.00E-04	RHIM	378-454	6.70E-07		-	
XP_010221010.1_Tinamus_guttatus	TRIF_NTD	4-160	1.20E-50	RHIM	368-418	7.20E-08		-	
XP_010288063.1_Phaethon_lepturus	TRIF_NTD	4-160	1.80E-54	TIR_2	445-564	1.80E-03	RHIM	648-705	1.60E-08
XP_010298283.1_Balearica_regulorum_gibbericeps	TRIF_NTD	4-160	1.00E-60	TIR_2	449-576	1.70E-03	RHIM	649-703	2.80E-05
XP_010365531.1_Rhinopithecus_roxellana	TRIF_NTD	1-148	6.80E-50	TIR_2	395-526	7.90E-03	RHIM	640-695	2.70E-12
XP_010410506.1_Corvus_cornix_cornix	TRIF_NTD	4-153	2.30E-46	TIR_2	427-550	2.60E-04	RHIM	610-674	1.70E-05
XP_010560547.1_Haliaeetus_leucocephalus	TRIF_NTD	4-160	8.60E-54	TIR_2	449-575	3.00E-03	RHIM	652-709	9.40E-09
XP_010633385.1_Fukomys_damarensis	TRIF_NTD	1-122	1.50E-22	RHIM	271-318	6.40E-08		-	
XP_010723390.1_Meleagris_gallopavo	TIR_2	322-448	6.70E-06	RHIM	547-597	4.30E-10		-	
XP_010736595.2_Larimichthys_crocea	TRIF_NTD	6-162	4.10E-45	TIR_2	353-481	1.10E-04		-	
XP_010774263.1_Notothenia_coriiceps	TRIF_NTD	6-162	1.30E-39	TIR_2	342-469	6.00E-07		-	
XP_010826467.1_Bison_bison_bison	TRIF_NTD	1-148	6.60E-52	RHIM	697-750	1.10E-09		-	
XP_010882521.3_Esox_lucius	TRIF_NTD	14-172	2.30E-31	TIR_2	395-522	4.40E-07		-	
XP_010965043.1_Camelus_bactrianus	TRIF_NTD	1-148	8.60E-49	TIR_2	391-527	1.60E-03	RHIM	647-708	1.80E-10
XP_010981627.1_Camelus_dromedarius	TRIF_NTD	1-148	8.60E-49	TIR_2	391-527	1.60E-03	RHIM	647-708	1.80E-10
XP_011235704.1_Ailuropoda_melanoleuca	TRIF_NTD	1-148	8.80E-48	TIR_2	454-590	4.60E-03	RHIM	630-666	1.90E-06
XP_011371861.1_Pteropus_vampyrus	TRIF_NTD	1-149	1.20E-54	RHIM	623-679	2.10E-11		-	
XP_011485063.1_Oryzias_latipes	TRIF_NTD	5-159	2.30E-38	TIR_2	347-475	4.60E-05		-	
XP_011594215.1_Aquila_chrysaetos_canadensis	TRIF_NTD	4-160	1.20E-53	TIR_2	448-574	3.00E-03	RHIM	641-708	3.20E-07
XP_011798868.1_Colobus_angolensis_palliatus	TRIF_NTD	1-148	2.40E-49	TIR_2	391-514	8.80E-03	RHIM	636-691	3.50E-12
XP_011822758.1_Mandrillus_leucophaeus	TRIF_NTD	1-148	1.90E-49	RHIM	641-696	1.80E-12		-	
XP_011928527.1_Cercocebus_atys	TRIF_NTD	1-148	6.60E-50	TIR_2	396-524	8.10E-03	RHIM	641-696	1.80E-12
XP_012291832.1_Aotus_nancymaae	TRIF_NTD	1-148	1.70E-48	TIR_2	393-524	6.50E-03	RHIM	638-681	3.30E-12
XP_012358815.1_Nomascus_leucogenys	TRIF_NTD	1-148	6.40E-50	TIR_2	376-503	6.00E-03	RHIM	621-676	6.00E-13
XP_012370522.1_Octodon_degus	TRIF_NTD	1-148	2.40E-40	TIR_2	393-522	9.90E-04	RHIM	603-702	5.50E-04
XP_012416085.1_Odobenus_rosmarus_divergens	TRIF_NTD	3-150	3.50E-48	DUF4335	592-651	9.20E-03	RHIM	655-734	5.10E-10
XP_012501678.1_Propithecus_coquereli	TRIF_NTD	1-151	5.00E-49	RHIM	608-683	1.90E-12		-	
XP_012680748.1_Clupea_harengus	TRIF_NTD	18-179	3.80E-29	TIR_2	381-510	2.80E-05		-	
XP_012714371.1_Fundulus_heteroclitus	TRIF_NTD	6-162	2.50E-46	TIR_2	357-485	9.90E-05		-	

XP_012810436.1_Xenopus_tropicalis	TRIF_NTD	34-160	3.30E-11	TIR_2	326-457	2.60E-05	RHIM	482-542	5.70E-05
XP_012863134.1_Echinops_telfairi	TRIF_NTD	1-146	4.60E-40	RHIM	620-667	2.50E-08		-	
XP_012878106.1_Dipodomys_ordii	TIR_2	287-416	8.50E-03	RHIM	512-568	1.10E-09		-	
XP_013050712.1_Anser_cygnoides_domesticus	TRIF_NTD	4-160	6.70E-47	TIR_2	444-575	1.20E-05	RHIM	661-718	4.10E-10
XP_013158110.1_Falco_peregrinus	TRIF_NTD	4-160	3.20E-53	TIR_2	452-578	2.80E-04	RHIM	632-711	4.90E-07
XP_013798144.1_Apteryx_australis_mantelli	TRIF_NTD	4-160	1.00E-53	TIR_2	446-572	6.60E-03	RHIM	664-721	6.30E-09
XP_013820713.2_Capra_hircus	TRIF_NTD	1-148	8.20E-52	RHIM	690-778	2.70E-08		-	
XP_013877135.1_Austrofundulus_limnaeus	TRIF_NTD	7-163	7.30E-40	TIR_2	340-468	1.50E-04		-	
XP_013926540.1_Thamnophis_sirtalis	TRIF_NTD	15-170	5.40E-58	TIR_2	379-498	8.00E-03	RHIM	577-630	9.00E-08
XP_014025434.1_Salmo_salar	TRIF_NTD	13-172	1.40E-33	TIR_2	394-526	4.60E-04		-	
XP_014133217.1_Falco_cherrug	TRIF_NTD	4-160	3.20E-53	TIR_2	452-578	2.80E-04	RHIM	632-711	2.30E-07
XP_014338163.1_Bos_mutus	TRIF_NTD	1-148	6.00E-52	RHIM	652-705	1.00E-09		-	
XP_014341573.1_Latimeria_chalumnae	TRIF_NTD	4-152	1.30E-38	TIR_2	416-542	8.80E-04		-	
XP_014460416.1_Alligator_mississippiensis	TRIF_NTD	4-161	2.10E-47	RHIM	632-679	9.50E-09		-	
XP_014698545.1_Equus_asinus	TRIF_NTD	2-149	5.40E-47	TIR_2	388-518	1.90E-03		-	
XP_014742629.1_Sturnus_vulgaris	TRIF_NTD	4-157	1.30E-45	TIR_2	432-556	1.10E-03	RHIM	599-653	1.20E-08
XP_014808292.1_Calidris_pugnax	TRIF_NTD	4-154	4.20E-50	TIR_2	441-567	6.10E-04	RHIM	610-691	6.20E-04
XP_014840262.1_Poecilia_mexicana	TRIF_NTD	6-159	1.20E-52	TIR_2	345-474	4.40E-06		-	
XP_014898090.1_Poecilia_latipinna	TRIF_NTD	6-159	2.40E-53	TIR_2	346-474	9.50E-06		-	
XP_015220763.1_Lepisosteus_oculatus	TRIF_NTD	4-152	4.00E-32	TIR_2	413-541	2.20E-05		-	
XP_015242175.1_Cyprinodon_variegatus	TRIF_NTD	6-159	3.10E-41	TIR_2	350-478	4.00E-06		-	
XP_015276078.1_Gekko_japonicus	TRIF_NTD	4-157	7.40E-46	TIR_2	393-521	5.10E-05	RHIM	609-657	2.60E-05
XP_015399660.1_Panthera_tigris_altaica	TRIF_NTD	1-148	1.10E-51		-			-	
XP_015507135.1_Parus_major	TRIF_NTD	4-149	1.40E-42	TIR_2	420-544	1.70E-03	RHIM	597-666	1.00E-07
XP_015741701.1_Coturnix_japonica	TRIF_NTD	4-158	2.00E-40	TIR_2	436-561	9.20E-05	RHIM	651-709	1.20E-09
XP_015812586.1_Nothobranchius_furzeri	TRIF_NTD	6-159	2.00E-42	TIR_2	336-464	5.30E-07		-	
XP_016000154.1_Rousettus_aegyptiacus	TRIF_NTD	1-149	1.40E-54	TIR_2	392-523	2.60E-03	RHIM	620-679	4.20E-10
XP_016053353.1_Miniopterus_natalensis	TRIF_NTD	1-149	6.80E-51	TIR_2	389-520	5.60E-04	RHIM	608-676	4.10E-11
XP_016085949.1_Sinocyclocheilus_grahami	TRIF_NTD	9-158	8.10E-40	TIR_2	324-457	5.10E-08		-	
XP_016362910.1_Sinocyclocheilus_anshuiensis	TRIF_NTD	9-158	8.30E-39	TIR_2	324-454	5.10E-08		-	
XP_016411308.1_Sinocyclocheilus_rhinocerous	TRIF_NTD	9-158	2.60E-40	TIR_2	324-454	2.90E-08		-	
XP_017277648.1_Kryptolebias_marmoratus	TRIF_NTD	6-162	1.60E-39	TIR_2	348-477	1.10E-04		-	
XP_017383886.1_Cebus_capucinus_imitator	TRIF_NTD	1-148	5.90E-50	TIR_2	394-522	9.30E-03	RHIM	639-682	1.10E-12
XP_017506473.1_Manis_javanica	TRIF_NTD	1-148	1.50E-50	TIR_2	399-530	6.00E-03	RHIM	629-683	2.70E-10
XP_017576226.1_Pygocentrus_nattereri	TIR_2	337-460	1.10E-05		-			-	
XP_017597972.1_Corvus_brachyrhynchos	TRIF_NTD	4-153	2.30E-46	TIR_2	427-550	2.60E-04	RHIM	610-674	1.70E-05
XP_017689117.1_Lepidothrix_coronata	TRIF_NTD	4-156	1.80E-53	TIR_2	432-555	1.70E-04		-	
XP_017702677.1_Rhinopithecus_bieti	TRIF_NTD	1-148	6.80E-50	TIR_2	395-526	7.80E-03	RHIM	640-695	2.70E-12
XP_018109628.1_Xenopus_laevis	TRIF_NTD	5-130	1.80E-11	TIR_2	280-408	3.40E-04	RHIM	435-494	3.00E-05
XP_018532364.1_Lates_calcarifer	TRIF_NTD	6-162	2.50E-41	TIR_2	327-455	4.50E-07		-	
XP_018615517.2_Scleropages_formosus	TRIF_NTD	16-171	6.80E-35	TIR_2	420-551	1.10E-06		-	
XP_018939138.1_Cyprinus_carpio	TRIF_NTD	9-158	1.70E-38	TIR_2	324-453	8.10E-08		-	
XP_019286450.1_Panthera_pardus	TRIF_NTD	1-148	3.40E-50	TIR_2	432-568	1.70E-03	RHIM	627-703	1.40E-09
XP_019368973.1_Gavialis_gangeticus	TRIF_NTD	4-161	2.30E-45	RHIM	627-675	1.90E-08		-	
XP_019406074.1_Crocodylus_porosus	TRIF_NTD	4-161	8.30E-47	RHIM	627-674	6.80E-09		-	
XP_019508889.1_Hipposideros_armiger	TRIF_NTD	3-151	8.00E-52	TIR_2	389-521	8.40E-04	RHIM	627-678	4.80E-10
XP_019634610.1_Branchiostoma_belcheri	TIR_2	206-333	6.70E-13		-			-	

XP_019712450.1_Hippocampus_comes	TRIF_NTD	51-207	3.10E-37	TIR_2	388-516	3.70E-07		-	
XP_019819974.1_Bos_indicus	TRIF_NTD	1-148	1.10E-51	RHIM	698-751	1.10E-09		-	
XP_019955201.1_Paralichthys_olivaceus	TRIF_NTD	6-162	2.40E-38	TIR_2	339-468	2.90E-05		-	
XP_020021442.1_Castor_canadensis	TRIF_NTD	1-148	5.60E-46	TIR_2	392-523	5.60E-03	RHIM	665-716	9.80E-11
XP_020139539.1_Microcebus_murinus	TRIF_NTD	1-152	3.80E-47	RHIM	679-732	6.50E-12		-	
XP_020347942.1_Oncorhynchus_kisutch	TRIF_NTD	13-174	1.80E-34	TIR_2	402-534	9.80E-04		-	
XP_020366239.1_Rhincodon_typus	TIR_2	64-193	8.20E-06	RHIM	312-362	7.80E-03		-	
XP_020471693.1_Monopterus_albus	TRIF_NTD	6-161	2.40E-39	TIR_2	343-471	4.00E-03		-	
XP_020489648.1_Labrus_bergylta	TRIF_NTD	6-162	2.20E-42	TIR_2	344-472	1.10E-06		-	
XP_020649920.1_Pogona_vitticeps	TRIF_NTD	4-158	3.00E-47	TIR_2	387-517	6.10E-04	RHIM	572-639	4.40E-04
XP_020769932.1_Odocoileus_virginianus_texanus	TRIF_NTD	1-148	9.50E-52	TIR_2	436-566	2.30E-03	RHIM	686-753	3.10E-09
XP_020778302.1_Boleophthalmus_pectinirostris	TRIF_NTD	6-157	1.70E-30	TIR_2	316-443	1.70E-04		-	
XP_020826901.1_Phascolarctos_cinereus	TRIF_NTD	1-154	1.40E-55	TIR_2	412-540	1.20E-04	RHIM	633-686	6.30E-08
XP_021004996.1_Mus_caroli	TRIF_NTD	1-148	6.50E-39	TIR_2	400-529	2.00E-03	RHIM	665-717	4.60E-11
XP_021074167.2_Mus_pahari	TRIF_NTD	72-219	3.20E-39	TIR_2	463-594	2.70E-03	RHIM	676-788	2.40E-07
XP_021234663.1_Numida_meleagris	TRIF_NTD	4-152	7.20E-41	TIR_2	429-555	1.30E-05	RHIM	628-704	3.80E-08
XP_021454523.1_Oncorhynchus_mykiss	TRIF_NTD	13-174	2.10E-34	TIR_2	395-527	9.60E-04		-	
XP_021488601.1_Meriones_unguiculatus	TRIF_NTD	1-148	1.60E-38	RHIM	637-728	2.90E-07		-	
XP_021560944.1_Neomonachus_schauinslandi	TRIF_NTD	3-150	8.40E-49	RHIM	666-752	4.40E-10		-	
XP_022057778.1_Acanthochromis_polyacanthus	TRIF_NTD	6-162	1.10E-43	TIR_2	356-485	2.10E-04		-	
XP_022381312.1_Enhydra_lutris_kenyoni	TRIF_NTD	3-150	2.40E-48	RHIM	681-724	6.10E-11		-	
XP_022412677.1_Delphinapterus_leucas	TRIF_NTD	1-148	2.10E-52	TIR_2	391-541	9.30E-03	RHIM	643-692	2.00E-10
XP_022611093.1_Seriola_dumerili	TRIF_NTD	6-162	2.70E-41	TIR_2	341-470	8.30E-06		-	
XP_023106646.1_Felis_catus	TRIF_NTD	1-148	7.40E-50	TIR_2	434-566	6.70E-03	RHIM	643-705	1.80E-09
XP_023124681.1_Amphiprion_ocellaris	TRIF_NTD	6-162	7.70E-44	TIR_2	356-484	6.70E-04		-	
XP_023261679.1_Seriola_lalandi_dorsalis	TRIF_NTD	6-162	1.00E-41	TIR_2	341-470	1.50E-05		-	
XP_023500240.1_Equus_caballus	TRIF_NTD	2-149	1.40E-47	TIR_2	388-519	1.80E-03	RHIM	647-698	4.20E-10
XP_023699379.1_Paramormyrops_kingsleyae	TRIF_NTD	19-178	3.50E-30	TIR_2	410-535	1.00E-06		-	
XP_023802262.1_Cyanistes_caeruleus	TRIF_NTD	4-149	3.80E-43	TIR_2	420-544	3.40E-03	RHIM	604-666	3.00E-08
XP_023854570.1_Salvelinus_alpinus	TRIF_NTD	13-172	1.40E-35	TIR_2	396-528	1.50E-03		-	
XP_024094075.1_Pongo_abelii	TRIF_NTD	1-148	1.20E-49	TIR_2	396-523	3.20E-03	RHIM	641-696	4.10E-12
XP_024130230.1_Oryzias_melastigma	TRIF_NTD	6-154	8.70E-39	TIR_2	332-461	2.60E-03		-	
XP_024239574.1_Oncorhynchus_tshawytscha	TRIF_NTD	13-174	1.50E-34	TIR_2	401-533	9.80E-04		-	
XP_024425012.1_Desmodus_rotundus	TRIF_NTD	1-149	1.20E-52	TIR_2	389-520	5.00E-03	RHIM	644-689	8.10E-12
XP_024601823.1_Neophocaena_asiaeorientalis_asiaeorientalis	TRIF_NTD	1-148	3.70E-52	TIR_2	383-514	2.00E-03	RHIM	635-684	5.30E-11
XP_025028501.1_Python_bivittatus	TRIF_NTD	15-169	1.60E-53	RHIM	591-641	2.70E-07		-	
XP_025052284.1_Alligator_sinensis	TRIF_NTD	4-161	9.00E-47	RHIM	635-676	5.00E-09		-	
XP_025225054.1_Theropithecus_gelada	TRIF_NTD	1-148	1.90E-49	TIR_2	397-527	1.50E-03	RHIM	635-697	2.40E-12
XP_025312834.1_Canis_lupus_dingo	TRIF_NTD	1-148	1.40E-48	RHIM	692-736	2.60E-10		-	
XP_025717171.1_Callorhinus_ursinus	TRIF_NTD	3-150	1.30E-48	RHIM	672-728	2.20E-11		-	
XP_025772161.1_Puma_concolor	TRIF_NTD	1-92	1.60E-29		-			-	
XP_025875158.1_Vulpes_vulpes	TRIF_NTD	1-148	1.60E-48	TIR_2	450-570	9.50E-03	RHIM	682-738	9.40E-11
XP_025890944.1_Nothoprocta_perdicaria	TRIF_NTD	4-153	2.00E-39	TIR_2	327-448	7.00E-03	RHIM	531-586	8.10E-08
XP_025922997.1_Apteryx_rowi	TRIF_NTD	4-160	1.00E-53	TIR_2	446-572	6.60E-03	RHIM	664-721	6.30E-09
XP_025961666.1_Dromaius_novaehollandiae	TRIF_NTD	4-160	3.70E-50	TIR_2	442-566	3.00E-03	RHIM	659-716	2.10E-07
XP_026006330.1_Astatotilapia_calliptera	TRIF_NTD	6-164	5.30E-39	TIR_2	337-467	2.00E-05		-	

XP_026102610.1_Carassius_auratus	TRIF_NTD	9-158	8.10E-39	TIR_2	324-455	4.70E-08		-	
XP_026170345.1_Mastacembelus_armatus	TRIF_NTD	6-162	1.30E-39	TIR_2	347-475	1.20E-05		-	
XP_026229899.1_Anabas_testudineus	TRIF_NTD	6-158	1.00E-35	TIR_2	350-478	1.90E-04		-	
XP_026260016.1_Urocitellus_parryii	TRIF_NTD	1-148	9.10E-49	TIR_2	381-518	3.10E-03	RHIM	602-659	1.70E-07
XP_026307783.1_Piliocolobus_tephrosceles	TRIF_NTD	1-148	9.80E-50	TIR_2	394-526	3.10E-03	RHIM	639-694	3.60E-12
XP_026336897.1_Ursus_arctos_horribilis	TRIF_NTD	1-148	3.00E-48	TIR_2	454-585	4.50E-03	RHIM	650-736	1.00E-10
XP_026510767.1_Terrapene_carolina_triunguis	TRIF_NTD	4-157	2.60E-51	TIR_2	406-534	3.20E-03	RHIM	628-685	1.40E-08
XP_026544024.1_Notechis_scutatus	TRIF_NTD	16-170	1.60E-59	RHIM	585-628	2.70E-06		-	
XP_026576138.1_Pseudonaja_textilis	TRIF_NTD	16-170	4.80E-60	RHIM	584-629	2.90E-07		-	
XP_026640203.1_Microtus_ochrogaster	TRIF_NTD	1-148	5.70E-39	TIR_2	398-527	1.10E-03	RHIM	639-732	5.60E-04
XP_026720904.1_Athene_cunicularia	TRIF_NTD	4-157	3.20E-47	TIR_2	443-570	1.70E-03	RHIM	646-700	6.30E-07
XP_026783416.1_Pangasianodon_hypophthalmus	TIR_2	247-378	2.30E-07		-			-	
XP_026888704.1_Electrophorus_electricus	TIR_2	249-379	2.10E-05		-			-	
XP_026904973.1_Acinonyx_jubatus	TRIF_NTD	1-148	9.50E-51	TIR_2	434-565	6.80E-03	RHIM	643-705	1.80E-09
XP_026941203.1_Lagenorhynchus_obliquidens	TRIF_NTD	1-148	1.70E-51	TIR_2	391-522	9.40E-03	RHIM	645-692	1.80E-10
XP_027015434.1_Tachysurus_fulvidraco	TIR_2	243-374	3.40E-04		-			-	
XP_027403126.1_Bos_indicus_x_Bos_taurus	TRIF_NTD	1-148	6.60E-52	RHIM	698-751	1.10E-09		-	
XP_027442601.1_Zalophus_californianus	TRIF_NTD	3-150	7.90E-48	RHIM	662-728	1.30E-11		-	
XP_027522403.1_Corapipo_altera	TRIF_NTD	4-156	5.10E-52	TIR_2	433-557	5.00E-05		-	
XP_027562012.1_Neopelma_chrysocephalum	TRIF_NTD	4-156	1.00E-53	TIR_2	432-552	1.40E-04		-	
XP_027580340.1_Pipra_filicauda	TRIF_NTD	4-150	2.20E-49	TIR_2	427-550	1.30E-04		-	
XP_027625410.1_Tupaia_chinensis	TRIF_NTD	1-55	9.60E-15	TIR_2	311-441	9.50E-04	RHIM	542-603	8.50E-10
XP_027706781.1_Vombatus_ursinus	TRIF_NTD	1-154	4.20E-55	TIR_2	412-539	4.20E-04	RHIM	638-686	1.60E-07
XP_027759422.1_Empidonax_traillii	TRIF_NTD	4-153	3.50E-48	TIR_2	420-539	6.10E-05	RHIM	590-669	1.40E-04
XP_027808270.1_Marmota_flaviventris	TRIF_NTD	48-195	6.80E-50	TIR_2	439-570	3.80E-03	RHIM	666-724	1.10E-09
XP_027877518.1_Xiphophorus_couchianus	TRIF_NTD	6-159	1.20E-49	TIR_2	346-475	6.30E-06		-	
XP_027982158.1_Eumetopias_jubatus	TRIF_NTD	3-150	1.10E-47	RHIM	671-728	4.10E-12		-	
XP_028284193.1_Parambassis_ranga	TRIF_NTD	7-157	1.20E-43	TIR_2	320-448	1.90E-06		-	
XP_028296624.1_Gouania_willdenowi	TRIF_NTD	10-158	7.20E-29	TIR_2	262-391	3.60E-04		-	
XP_028375832.1_Phyllostomus_discolor	TRIF_NTD	1-149	1.60E-52	RHIM	638-693	5.00E-11		-	
XP_028449138.1_Perca_flavescens	TRIF_NTD	6-162	4.10E-40	TIR_2	353-483	6.10E-05		-	
XP_028569816.1_Podarcis_muralis	TRIF_NTD	101-254	5.70E-45	TIR_2	490-619	1.50E-03	RHIM	691-740	7.40E-07
XP_028618280.1_Grammomys_surdaster	TRIF_NTD	1-148	4.00E-38	TIR_2	398-533	1.40E-03	RHIM	611-687	3.30E-08
XP_028672432.1_Erpetoichthys_calabaricus	TRIF_NTD	2-154	6.40E-34	TIR_2	367-496	1.40E-06	RHIM	589-645	2.50E-03
XP_028715543.1_Peromyscus_leucopus	TRIF_NTD	1-148	4.40E-40	TIR_2	401-532	1.00E-03	RHIM	614-694	4.20E-08
XP_028856297.1_Denticeps_clupeoides	TRIF_NTD	11-155	1.00E-18	TIR_2	331-465	2.00E-07		-	
XP_028906958.1_Ornithorhynchus_anatinus	TRIF_NTD	2-123	1.40E-19	TIR_2	362-491	6.90E-04	RHIM	551-606	1.50E-09
XP_028988652.1_Betta_splendens	TRIF_NTD	6-161	1.10E-34	TIR_2	338-464	1.50E-03		-	
XP_029075017.1_Monodon_monoceros	TRIF_NTD	1-148	3.80E-52	RHIM	643-692	2.00E-10		-	
XP_029141819.1_Protobothrops_mucrosquamatus	TRIF_NTD	4-158	9.20E-56	TIR_2	239-364	5.00E-03	RHIM	428-477	1.10E-07
XP_029308550.1_Cottoperca_gobio	TRIF_NTD	6-162	7.70E-41	TIR_2	340-468	1.30E-05		-	
XP_029380951.1_Echeneis_naucrates	TRIF_NTD	6-162	2.30E-43	TIR_2	329-457	2.00E-04		-	
XP_029470442.1_Rhinatrema_bivittatum	TRIF_NTD	9-159	4.00E-32	TIR_2	401-523	5.20E-05	RHIM	651-692	1.90E-05
XP_029541726.1_Oncorhynchus_nerka	TRIF_NTD	13-174	2.00E-34	TIR_2	402-534	9.80E-04		-	
XP_029607373.1_Salmo_trutta	TRIF_NTD	13-172	1.20E-35	TIR_2	393-525	4.60E-04		-	
XP_029773543.1_Suricata_suricatta	TRIF_NTD	1-148	1.90E-48	TIR_2	415-546	2.50E-03	RHIM	599-686	2.00E-09

XP_029931358.1_Myripristis_murdjan	TRIF_NTD	6-164	1.90E-39	TIR_2	389-517	1.20E-04		-	
XP_029970829.1_Salarias_fasciatus	TRIF_NTD	2-155	2.10E-34	TIR_2	331-459	8.60E-03		-	
XP_030015455.1_Sphaeramia_orbicularis	TRIF_NTD	6-162	3.70E-40	TIR_2	349-478	1.50E-07		-	
XP_030073778.1_Microcaecilia_unicolor	TRIF_NTD	17-171	1.00E-37	TIR_2	412-538	6.40E-06	RHIM	604-692	2.30E-03
XP_030148660.1_Lynx_canadensis	TRIF_NTD	1-148	7.90E-50	TIR_2	426-557	6.30E-03	RHIM	635-697	1.80E-09
XP_030220008.1_Gadus_morhua	TRIF_NTD	6-161	1.40E-30	TIR_2	346-478	1.20E-06		-	
XP_030257680.1_Sparus_aurata	TRIF_NTD	6-160	9.50E-41	TIR_2	328-456	2.00E-06		-	
XP_030322340.1_Calypte_anna	TRIF_NTD	4-159	4.50E-46	TIR_2	446-575	1.40E-04	RHIM	649-697	6.30E-09
XP_030365526.1_Strigops_habroptila	TRIF_NTD	4-156	1.30E-48	TIR_2	434-560	1.00E-03	RHIM	626-676	2.90E-08
XP_030396462.1_Gopherus_evgoodei	TRIF_NTD	4-157	2.60E-50	TIR_2	409-535	1.20E-04	RHIM	628-686	3.60E-08
XP_030608078.1_Archocentrus_centrarchus	TRIF_NTD	17-173	1.80E-42		-			-	

TRAM orthologues

Node ID	domain	domain boundary	e-value
2M1W_A	TIR_2	12-141	4.30E-09
AFN27530.1_Capra_hircus	TIR_2	78-206	4.40E-09
ETE60420.1_Ophiophagus_hannah	TIR_2	61-199	1.00E-07
NP_001039921.1_Bos_taurus	TIR_2	78-206	2.10E-08
NP_001102360.1_Rattus_norvegicus	TIR_2	78-206	1.40E-07
NP_001182133.1_Pan_troglodytes	TIR_2	81-209	1.90E-08
NP_001182430.1_Macaca_mulatta	TIR_2	81-209	1.90E-08
NP_001189783.1_Equus_caballus	TIR_2	84-212	1.30E-08
NP_001191270.1_Pongo_abelii	TIR_2	81-209	1.90E-08
NP_001191272.1_Callithrix_jacchus	TIR_2	80-208	1.90E-08
NP_001191275.1_Oryctolagus_cuniculus	TIR_2	87-216	4.30E-08
NP_001191276.1_Monodelphis_domestica	TIR_2	73-197	1.50E-07
NP_001191280.1_Sus_scrofa	TIR_2	83-212	2.00E-08
NP_001191386.1_Ornithorhynchus_anatinus	TIR_2	85-204	1.10E-07
NP_001269274.1_Gorilla_gorilla	TIR_2	81-209	1.90E-08
NP_001272750.1_Macaca_fascicularis	TIR_2	81-209	1.90E-08
NP_001279313.1_Callorhinchus_milii	TIR_2	73-200	1.80E-08
NP_067681.1_Homo_sapiens	TIR_2	81-209	1.90E-08
NP_775570.1_Mus_musculus	TIR_2	78-206	5.90E-08
OBS82825.1_Neotoma_lepida	TIR_2	65-190	5.80E-08
TEA40782.1_Sousa_chinensis	TIR_2	81-209	3.00E-09
TFK12723.1_Platysternon_megacephalum	TIR_2	67-213	7.80E-09
XP_003259902.1_Nomascus_leucogenys	TIR_2	80-209	2.50E-08
XP_003788954.1_Otolemur_garnettii	TIR_2	81-209	5.40E-08
XP_004010232.2_Ovis_aries	TIR_2	75-203	2.50E-09
XP_004267517.1_Orcinus_orca	TIR_2	81-209	3.00E-09
XP_004599906.1_Ochotona_princeps	TIR_2	31-160	3.90E-08
XP_004618241.1_Sorex_araneus	TIR_2	61-190	6.70E-08
XP_004651622.1_Jaculus_jaculus	TIR_2	81-209	1.30E-08
XP_004686551.1_Condylura_cristata	TIR_2	80-231	4.10E-10
XP_004706631.1_Echinops_telfairi	TIR_2	85-214	3.70E-07
XP_004759121.1_Mustela_putorius_furo	TIR_2	90-218	1.10E-08
XP_005244703.1_Falco_peregrinus			
XP_005300191.1_Chrysemys_picta_bellii	TIR_2	67-196	7.10E-09
XP_005356131.1_Microtus_ochrogaster	TIR_2	77-206	1.80E-07
XP_005447542.1_Falco_cherrug			
XP_005857418.1_Myotis_brandtii	TIR_2	80-208	8.20E-09
XP_005902009.1_Bos_mutus	TIR_2	108-230	1.20E-05
XP_006047327.1_Bubalus_bubalis	TIR_2	78-207	2.10E-08
XP_006081198.1_Myotis_lucifugus	TIR_2	80-203	1.20E-08
XP_006736938.1_Leptonychotes_weddellii	TIR_2	84-212	7.30E-09
XP_006755335.1_Myotis_davidii	TIR_2	80-207	8.60E-09
XP_006870605.1_Chrysochloris_asiatica	TIR_2	85-213	1.60E-07
XP_006886760.1_Elephantulus_edwardii	TIR_2	81-204	4.70E-07

XP_006913357.1_Pteropus_alecto	TIR_2	80-209	1.60E-08
XP_006927597.1_Felis_catus	TIR_2	83-211	6.10E-09
XP_006977723.1_Peromyscus_maniculatus_bairdii	TIR_2	79-207	2.60E-08
XP_007055469.2_Chelonia_mydas	TIR_2	71-200	3.50E-09
XP_007121541.1_Physeter_catodon	TIR_2	81-209	3.90E-09
XP_007192181.1_Balaenoptera_acutorostrata_scammoni	TIR_2	81-209	4.20E-09
XP_007442151.1_Python_bivittatus	TIR_2	61-199	5.20E-07
XP_007451276.1_Lipotes_vexillifer	TIR_2	81-209	3.00E-09
XP_007952719.1_Orycteropus_afer_afer	TIR_2	81-209	6.20E-07
XP_008012375.1_Chlorocebus_sabaeus	TIR_2	81-209	1.90E-08
XP_008070631.1_Carlito_syrichta	TIR_2	79-207	1.50E-08
XP_008142680.1_Eptesicus_fuscus	TIR_2	80-207	9.30E-09
XP_008586847.1_Galeopterus_variegatus	TIR_2	80-209	1.20E-07
XP_008707699.1_Ursus_maritimus	TIR_2	71-199	1.60E-08
XP_008937694.1_Merops_nubicus			
XP_008950312.1_Pan_paniscus	TIR_2	81-209	1.90E-08
XP_009072374.1_Acanthisitta_chloris			
XP_009207178.1_Papio_anubis	TIR_2	81-209	1.90E-08
XP_009282474.1_Aptenodytes_forsteri			
XP_009319388.1_Pygoscelis_adeliae			
XP_009467472.1_Nipponia_nippon			
XP_009476711.1_Pelecanus_crispus			
XP_009512440.1_Phalacrocorax_carbo			
XP_009580203.1_Fulmarus_glacialis	RVT_1	89-191	0.0032
XP_009634862.1_Egretta_garzetta			
XP_009707473.1_Cariama_cristata			
XP_009872473.1_Apaloderma_vittatum			
XP_009892132.1_Charadrius_vociferus			
XP_009898099.1_Picoides_pubescens			
XP_009927540.1_Haliaeetus_albicilla	TIR_2	9-134	0.0023
XP_009930508.1_Opisthocomus_hoazin			
XP_010077229.1_Pterocles_gutturalis			
XP_010113783.1_Chlamydotis_macqueenii			
XP_010145478.1_Eurypyga_helias			
XP_010282756.1_Phaethon_lepturus			
XP_010304648.1_Balearica_regulorum_gibbericeps			
XP_010337966.1_Saimiri_boliviensis_boliviensis	TIR_2	80-208	2.00E-08
XP_010355222.1_Rhinopithecus_roxellana	TIR_2	80-208	1.50E-08
XP_010612439.1_Fukomys_damarensis	TIR_2	81-210	1.60E-08
XP_010860382.1_Bison_bison_bison	TIR_2	78-206	1.60E-08
XP_010969814.1_Camelus_bactrianus	TIR_2	82-210	1.60E-08
XP_011225882.1_Ailuropoda_melanoleuca	TIR_2	80-208	1.50E-08
XP_011356556.1_Pteropus_vampyrus	TIR_2	80-209	1.60E-08
XP_011599118.1_Aquila_chrysaetos_canadensis			
XP_011819052.1_Colobus_angolensis_palliatus	TIR_2	81-209	1.90E-08
XP_012294094.1_Aotus_nancymaae	TIR_2	80-208	2.00E-08
XP_012372212.1_Octodon_degus	TIR_2	80-209	1.60E-08

XP_012399572.1_Sarcophilus_harrisii	TIR_2	71-197	3.70E-09
XP_012418625.1_Odobenus_rosmarus_divergens	TIR_2	79-206	5.50E-09
XP_012515001.1_Propithecus_coquereli	TIR_2	82-210	8.00E-08
XP_012619167.1_Microcebus_murinus	TIR_2	82-210	5.70E-08
XP_012871538.1_Dipodomys_ordii	TIR_2	80-208	1.30E-08
XP_012968196.1_Mesocricetus_auratus	TIR_2	79-208	4.50E-07
XP_013011597.1_Cavia_porcellus	TIR_2	74-203	3.90E-09
XP_013053059.1_Anser_cygnoides_domesticus			
XP_013220277.1_Ictidomys_tridecemlineatus	TIR_2	81-209	3.50E-08
XP_013366174.1_Chinchilla_lanigera	TIR	53-150	1.20E-06
XP_013912448.1_Thamnophis_sirtalis	TIR_2	62-191	1.10E-07
XP_014434708.1_Pelodiscus_sinensis	TIR_2	65-195	4.50E-08
XP_014447016.1_Tupaia_chinensis	TIR_2	90-218	2.10E-08
XP_014652124.1_Ceratotherium_simum_simum	TIR_2	81-209	1.30E-08
XP_014697198.1_Equus_asinus	TIR_2	84-212	1.30E-08
XP_014797954.1_Calidris_pugnax			
XP_014932168.1_Acinonyx_jubatus	TIR_2	83-211	6.10E-09
XP_015094364.1_Vicugna_pacos	TIR_2	82-211	1.60E-08
XP_015271788.1_Gekko_japonicus	TIR_2	68-214	6.10E-06
XP_015344087.1_Marmota_marmota_marmota	TIR_2	81-209	3.50E-08
XP_015675321.1_Protobothrops_mucrosquamatus	TIR_2	61-198	2.90E-08
XP_016002930.1_Rousettus_aegyptiacus	TIR_2	80-208	1.50E-08
XP_016080423.1_Miniopterus_natalensis	TIR_2	83-211	2.90E-09
XP_016850341.1_Anolis_carolinensis	TIR_2	68-197	2.10E-05
XP_017357511.1_Cebus_capucinus_imitator	TIR_2	80-208	1.90E-08
XP_017533085.1_Manis_javanica	TIR_2	83-211	5.90E-08
XP_017651625.1_Nannospalax_galili	TIR_2	78-206	9.10E-09
XP_017744484.1_Rhinopithecus_bieti	TIR_2	81-209	1.50E-08
XP_018119788.1_Xenopus_laevis	TIR_2	26-157	1.00E-10
XP_018415358.1_Nanorana_parkeri	TIR_2	57-189	1.30E-08
XP_019340091.1_Alligator_mississippiensis	TIR_2	78-207	7.60E-07
XP_019372527.1_Gavialis_gangeticus	TIR_2	78-207	8.50E-07
XP_019410568.1_Crocodylus_porosus	TIR_2	78-207	4.10E-07
XP_019506796.1_Hipposideros_armiger	TIR_2	80-208	6.30E-08
XP_020041793.1_Castor_canadensis	TIR_2	79-208	1.60E-08
XP_020748203.1_Odocoileus_virginianus_texanus	TIR_2	78-206	2.10E-08
XP_020823005.1_Phascolarctos_cinereus	TIR_2	73-201	1.50E-07
XP_021006287.1_Mus_caroli	TIR_2	78-206	5.90E-08
XP_021070064.1_Mus_pahari	TIR_2	98-226	7.70E-08
XP_021508228.1_Meriones_unguiculatus	TIR_2	78-203	1.10E-07
XP_021557894.1_Neomonachus_schauinslandi	TIR_2	84-211	5.30E-09
XP_022376890.1_Enhydra_lutris_kenyoni	TIR_2	89-217	1.10E-08
XP_022446558.1_Delphinapterus_leucas	TIR_2	81-209	2.80E-09
XP_023404744.1_Loxodonta_africana	TIR_2	78-206	3.00E-07
XP_023447126.1_Dasypus_novemcinctus	TIR_2	95-223	5.80E-09
XP_023587721.1_Trichechus_manatus_latirostris	TIR_2	79-207	1.70E-07
XP_024064535.2_Terrapene_carolina_triunguis	TIR_2	67-196	7.10E-09

XP_024419068.1_Desmodus_rotundus	TIR_2	80-208	1.80E-07
XP_024599102.1_Neophocaena_asiaeorientalis_asiaeorient	TIR_2	81-209	2.80E-09
XP_025716559.1_Callorhinus_ursinus	TIR_2	79-206	5.50E-09
XP_025780880.1_Puma_concolor	TIR_2	83-211	6.10E-09
XP_026271260.1_Urocitellus_parryii	TIR_2	81-209	3.60E-08
XP_026302779.1_Piliocolobus_tephrosceles	TIR_2	81-209	2.00E-08
XP_026363570.1_Ursus_arctos_horribilis	TIR_2	80-208	1.50E-08
XP_026529181.1_Notechis_scutatus	TIR_2	61-197	9.10E-08
XP_026575965.1_Pseudonaja_textilis	TIR_2	61-197	1.10E-07
XP_027256662.1_Cricetulus_griseus	TIR_2	100-228	4.70E-07
XP_027460744.1_Zalophus_californianus	TIR_2	79-206	5.50E-09
XP_027564076.1_Neopelma_chrysocephalum	TIR_2	45-179	0.0063
XP_027730559.1_Vombatus_ursinus	TIR_2	78-206	1.20E-07
XP_027796854.1_Marmota_flaviventris	TIR_2	81-209	3.50E-08
XP_027948430.1_Eumetopias_jubatus	TIR_2	79-206	5.50E-09
XP_028604507.1_Podarcis_muralis	TIR_2	68-197	9.30E-06
XP_028632962.1_Grammomys_surdaster	TIR_2	78-206	5.70E-08
XP_029427129.1_Rhinatrema_bivittatum	TIR_2	83-214	8.60E-10
XP_029797434.1_Suricata_suricatta	TIR_2	80-208	5.20E-09
XP_029860006.1_Aquila_chrysaetos_chrysaetos	TIR_2	89-212	0.0039
XP_030049363.1_Microcaecilia_unicolor	TIR_2	76-209	1.20E-09
XP_030084069.1_Serinus_canaria			
XP_030172679.1_Lynx_canadensis	TIR_2	83-211	6.10E-09
XP_030421630.1_Gopherus_evgoodei	TIR_2	67-196	6.80E-09
